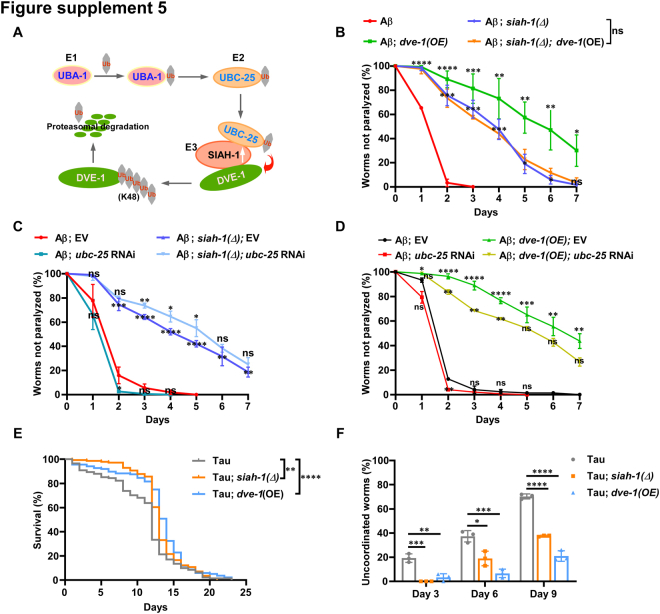# Inactivation of SIAH-1 E3 ligase attenuates Aβ toxicity by suppressing ubiquitin-dependent DVE-1 degradation in *Caenorhabditis elegans* models of Alzheimer’s disease

**DOI:** 10.1016/j.jbc.2025.110226

**Published:** 2025-05-09

**Authors:** Lihua Sun, Jiahui Liu, Menghan Lu, Yingying Zhou, Shuqi Guo, Zhipeng Qin, Zekun Wang, Xiaojuan Sun

**Affiliations:** 1 The Zhongzhou Laboratory for Integrative Biology, School of Basic Medical Sciences, Henan University, Kaifeng, Henan, PR China; 2 Joint National Laboratory for Antibody Drug Engineering, School of Medicine, Henan University, Kaifeng, PR China

**Keywords:** *C. elegans*, E3 ubiquitin ligase SIAH-1, Aβ toxicity, UPR^mt^, DVE-1, proteasomal degradation

## Abstract

The mitochondrial unfolded protein response (UPR^mt^), an evolutionarily conserved proteostasis pathway, plays a critical role in the pathogenesis of Alzheimer's disease (AD), characterized by amyloid-**β** peptide (A**β**) aggregation. Although the transcription factor DVE-1 regulates UPR^mt^ activation in *Caenorhabditis elegans* and has been implicated in A**β** pathology, its regulatory mechanisms under AD-like conditions remain unclear. Here, using the classical *C. elegans* muscle-specific AD model (CL2006 strain), we observed UPR^mt^ induction in young adults despite paradoxical depletion of DVE-1 protein concurrent with elevated *dve-1* transcript levels. Through integrated genetic and biochemical analyses, we identified SIAH-1, a conserved E3 ubiquitin ligase that partners with the E2 enzyme UBC-25 to interact with DVE-1 and mediate its K48-linked polyubiquitination, as targeting DVE-1 for proteasomal degradation. Disruption of SIAH-1 E3 ubiquitin ligase function or overexpression of DVE-1 significantly reduced A**β** toxicity in both the muscle-expressed A**β** (CL2006) and neuronal A**β** models *(gnaIs2)*. These interventions concurrently suppressed Aβ aggregation in the heat shock-inducible A**β** aggregation model (*xchIs15*). Mechanistically, this protective effect was associated with restored mitochondrial homeostasis, as evidenced by MitoTracker Red staining and TOMM-20::mCherry fluorescence imaging in muscle-expressed A**β** animals. These assays demonstrated that A**β** accumulation compromises mitochondrial integrity, a phenotype markedly rescued in *siah-1* deletion mutants and DVE-1-overexpressing strains. Collectively, these findings establish the SIAH-1/DVE-1 axis as a conserved proteostasis regulator and highlight ubiquitin-dependent mitochondrial quality control as a potential therapeutic target for AD and related proteopathies.

Alzheimer's disease, the most prevalent form of dementia in the elderly, is characterized by the accumulation of Aβ plaques and progressive neurodegeneration (1). Despite decades of research, no cure exists, underscoring the urgent need to unravel the molecular mechanisms underlying Aβ proteotoxicity and identify novel therapeutic targets. Mitochondrial dysfunction is closely linked to Aβ-induced pathogenesis in AD (2, 3), and enhancing mitochondrial proteostasis to counteract Aβ toxicity has emerged as a promising target for therapeutic development (4, 5). Transcripts of several mitochondrial unfolded protein response (UPR^mt^) genes are upregulated in patients with early AD (6), and UPR^mt^ activation has also been observed in both mouse and cell models of mild AD pathogenesis (7) as well as in the *Caenorhabditis elegans* muscle-expressed Aβ model (GMC101 strain) (5). Furthermore, Aβ_42_ has been shown to directly interact with LONP1, a matrix ATP-dependent mitochondrial protease, disrupting its assembly and activity, thereby impairing mitochondrial proteostasis. Overexpression of LONP1 has been shown to rescue Aβ-induced toxicity in a mouse AD model (8), highlighting a potential interplay between Aβ toxicity and mitochondrial proteostasis.

The UPR^mt^ is a key evolutionarily conserved quality control pathway that regulates mitochondrial proteostasis (9, 10, 11). In *C. elegans*, the transcription factor (TF) DVE-1, homologous to human SATB1/SATB2, operates in parallel with another TF, ATFS-1, to regulate the UPR^mt^ (12, 13, 14). Upon mitochondrial perturbation, DVE-1 translocates from the cytosol to the nucleus to induce chromatin reorganization and promote the transcriptional expression of crucial genes in response to mitochondrial stress (15, 16). While several factors that coordinate with DVE-1 to modulate the UPR^mt^ have been identified (15, 16, 17, 18), the turnover of DVE-1 and its regulation under pathological conditions like AD have not been explored.

The ubiquitin–proteasome system (UPS), the primary protein quality control machinery in eukaryotic cells, degrades Aβ but is itself inhibited by Aβ, which blocks 26S proteasome activity (19). E3 ubiquitin ligases, guided by E1 activating enzyme and E2 conjugating enzyme, determine substrate specificity in UPS-mediated degradation (20). Intriguingly, mammalian SIAH1, an E3 ligase implicated in Parkinson’s disease (PD) and enriched in Lewy bodies (21), has not been studied in the context of UPR^mt^ regulation or Aβ proteotoxicity.

In this study, using the classical *C. elegans* muscle-expressed Aβ model (CL2006), which exhibits adult-onset progressive paralysis that is exacerbated by elevated temperature (22, 23), we identified SIAH-1, a conserved RING-finger E3 ligase, as the critical regulator of DVE-1 stability. We demonstrated that Aβ upregulates SIAH-1, which partners with the E2 enzyme UBC-25 to promote K48-linked polyubiquitination and proteasomal degradation of DVE-1. Genetic ablation of *siah-1* or overexpression of DVE-1 mitigated Aβ aggregation and associated toxicity across multiple *C. elegans* AD models. Mechanistically, Aβ accumulation in CL2006 disrupted mitochondrial integrity, as shown by MitoTracker Red and TOMM-20::mCherry assays, a phenotype significantly rescued in *siah-1* deletion mutants or DVE-1-overexpressing strains. These findings reveal a self-reinforcing cycle, wherein Aβ exacerbates mitochondrial dysfunction by destabilizing DVE-1 *via* SIAH-1.

Taken together, our work delineates the SIAH-1/DVE-1 axis as a critical nexus in proteostasis regulation, providing both mechanistic insights into neurodegenerative cascades and actionable targets for enhancing cellular stress resilience in AD and related disorders.

## Results

### A**β** triggers proteasomal degradation of DVE-1

**Figure S1:**
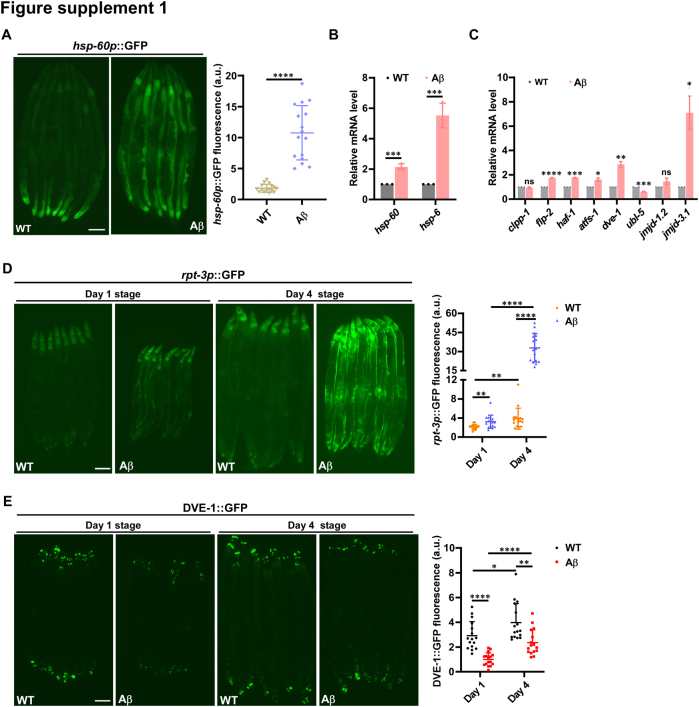


The *C. elegans* transgenic strain CL2006 *dvIs2(unc-54p::*Aβ_1-42_*)*, which expresses human Aβ peptide under the muscle-specific promoter of *unc-54*, is widely used to study Aβ toxicity in AD research (24). We found that CL2006 young adults exhibited activated UPR^mt^, as indicated by a reporter that expressed GFP from the *hsp-60* promoter *(hsp-60p::gfp)* (Fig. S1*A*), mirroring the increased UPR^mt^ observed in early-stage AD patients (6). This activation was corroborated by increased mRNA levels of the UPR^mt^ markers *hsp-60* and *hsp-6* in CL2006 worms (Fig. S1*B*). Furthermore, transcriptional analysis revealed upregulation of key UPR^mt^-related genes, including *flp-2*, *haf-1*, *atfs-1*, *dve-1*, and *jmjd-3.1* (Fig. S1*C*).

**Figure 1:**
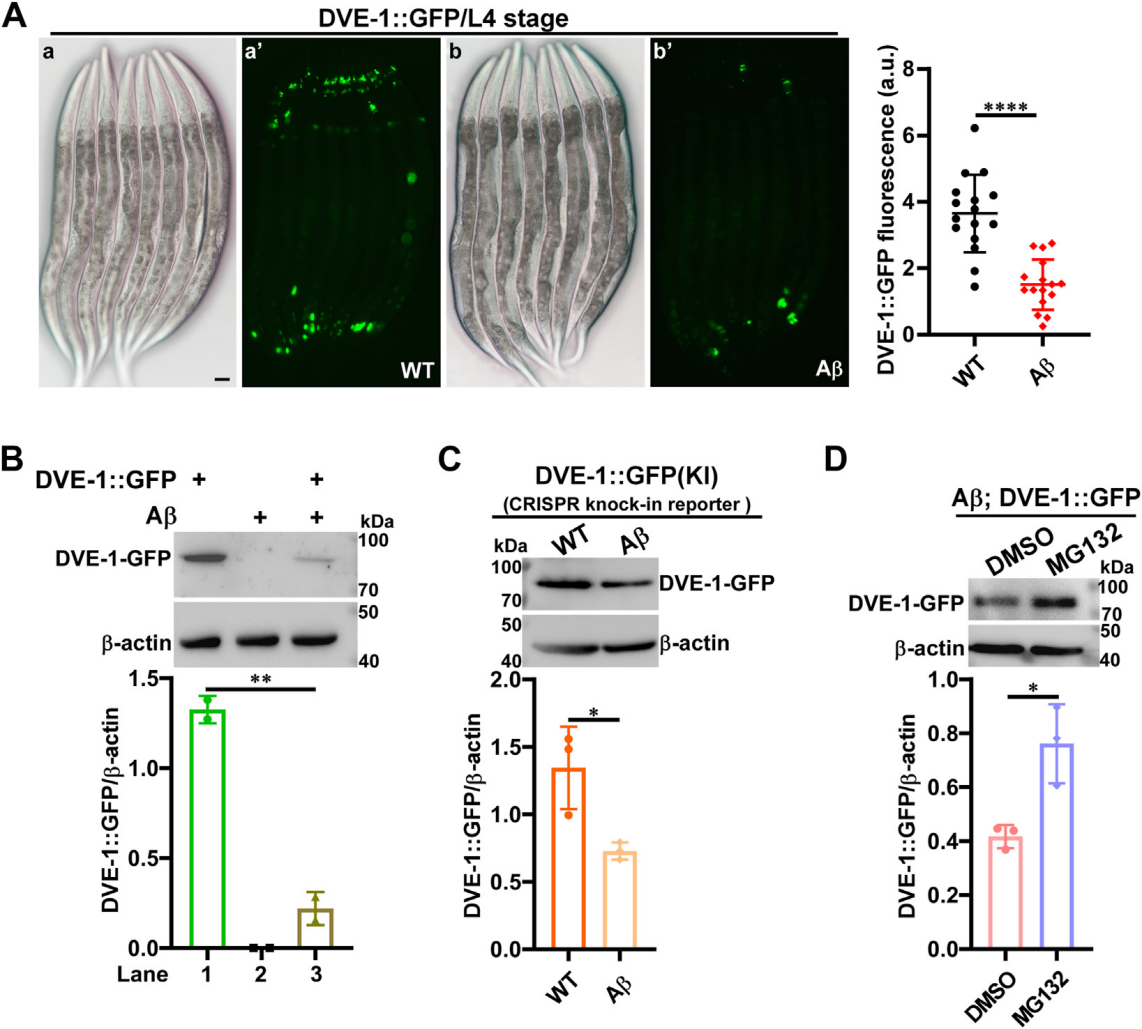
**DVE-1::GFP is reduced in the presence of Aβ.***A*, representative photomicrographs of *C. elegans* expressing DVE-1::GFP at the L4 stage are shown. (a-a’) DIC and fluorescence images of *zcIs39(dve-1p::dve-1::gfp)* worms, and DVE-1::GFP is predominantly expressed in the head and intestinal nuclei; (b-b’) images of DVE-1::GFP in CL2006 *dvIs2(unc-54p::*Aβ_1-42_*)* animals. Fluorescence images were captured under identical conditions. Scale bar: 20 μm. Quantification of DVE-1::GFP fluorescence intensity reveals a significant reduction in *dvIs2* animals compared to wild-type controls (n ≥ 15 worms per group. Unpaired *t*-tests, ∗∗∗∗*p* < 0.0001). *B*, Western blot analysis of DVE-1::GFP and β-actin (loading control) in day 1 adult worms of the following strains: *zcIs39* (lane 1), *dvIs2* (lane 2), and *dvIs2; zcIs39* (lane 3) worms. Quantification of DVE-1::GFP protein levels, normalized to β-actin in each group, demonstrated a significant decrease in *dvIs2; zcIs39* animals compared to controls (∗∗*p* < 0.01). *C*, Western blot analysis was performed to detect DVE-1::GFP levels in *syb1984*, a CRISPR-Cas9 generated GFP insertion line (denoted as DVE-1::GFP(KI)), and *dvIs2; syb1984* worms. β-actin serves as a loading control. Quantification of DVE-1::GFP protein levels, normalized to β-actin levels in each group, revealed a statistically significant reduction in *dvIs2; syb1984* worms compared to the *syb1984* controls (∗*p* < 0.05). *D*, Western blot analysis of DVE-1::GFP and β-actin in day 1 adult *dvIs2; zcIs39* worms treated with DMSO or 10 μM MG132. DVE-1::GFP levels, normalized to β-actin, increased significantly in MG132-treated worms compared to DMSO controls (∗*p* < 0.05).

Interestingly, despite elevated *dve-1* mRNA levels in CL2006 worms (Fig. S1*C*), we observed significantly decreased fluorescence intensity of DVE-1::GFP in the presence of Aβ using the transgenic strain expressing DVE-1::GFP (*zcIs39*), both in the head and in the intestine (Fig. 1*A*). We then performed Western blot and confirmed a significant decrease in DVE-1-GFP protein levels in the presence of Aβ (Fig. 1*B*).

We further investigated this phenomenon using the *syb1984* strain, a CRISPR-Cas9-generated *dve-1::gfp* reporter with GFP knocked into the endogenous *dve-1* C-terminus (denoted as DVE-1::GFP(KI)). A similar reduction in endogenous DVE-1::GFP protein levels *via* Western blot was observed in Aβ; DVE-1::GFP(KI) worms (Fig. 1*C*), consistent with that in Aβ; DVE-1::GFP worms (Fig. 1*B*).

Human Aβ has been shown to induce proteasome dysfunction in *C. elegans*, triggering a compensatory response *via* SKN-1A/Nrf1-mediated activation of proteasome subunit expression, as demonstrated by a reporter that expressed GFP from the *rpt-3* promoter (*rpt-3p::gfp*) (25). In the presence of Aβ, transcription of the proteasome reporter gene *rpt-3* increased with age (Fig. S1*D*), indicating proteasome function perturbation. Concurrently, fluorescence in Aβ; DVE-1::GFP worms was elevated at Day 4 compared to Day 1 (Fig. S1*E*), suggesting that DVE-1::GFP is likely degraded by the proteasome in the presence of Aβ. We then treated young adult Aβ; DVE-1::GFP worms with MG132, a commonly used proteasome inhibitor, and observed a significant restoration of DVE-1::GFP compared to the DMSO control treatment (Fig. 1*D*). Collectively, these results suggest that Aβ may induce proteasomal degradation of DVE-1.

### SIAH-1 mediates the degradation of DVE-1

**Figure S2:**
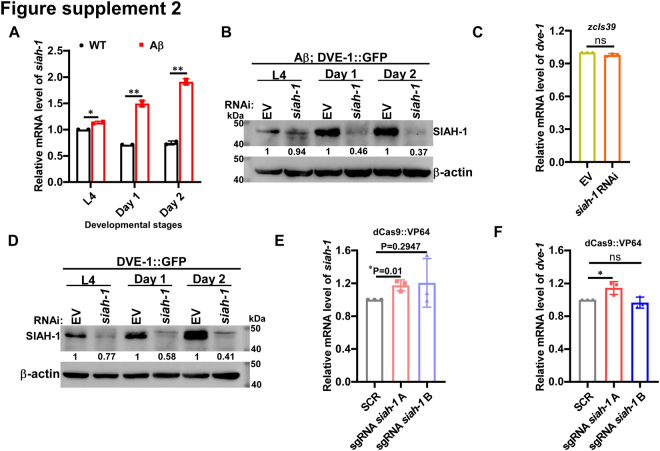


To elucidate the mechanism of DVE-1 downregulation in the presence of Aβ, we performed mRNA sequencing (mRNA-seq) on synchronized young adult muscle-expressed Aβ (CL2006) worms and wild-type worms. This analysis identified 98 downregulated genes and 416 upregulated genes (Supplementary Excel file 1). Among the upregulated genes, we found *siah-1*, encoding an evolutionarily conserved E3 ubiquitin protein ligase. Its homolog, Siah1, is known to be involved in the proteasomal degradation of target proteins (26, 27, 28, 29, 30). We validated the increased *siah-1* mRNA levels in CL2006 worms at different developmental stages compared to wild-type controls (Fig. S2*A*).

**Figure 2:**
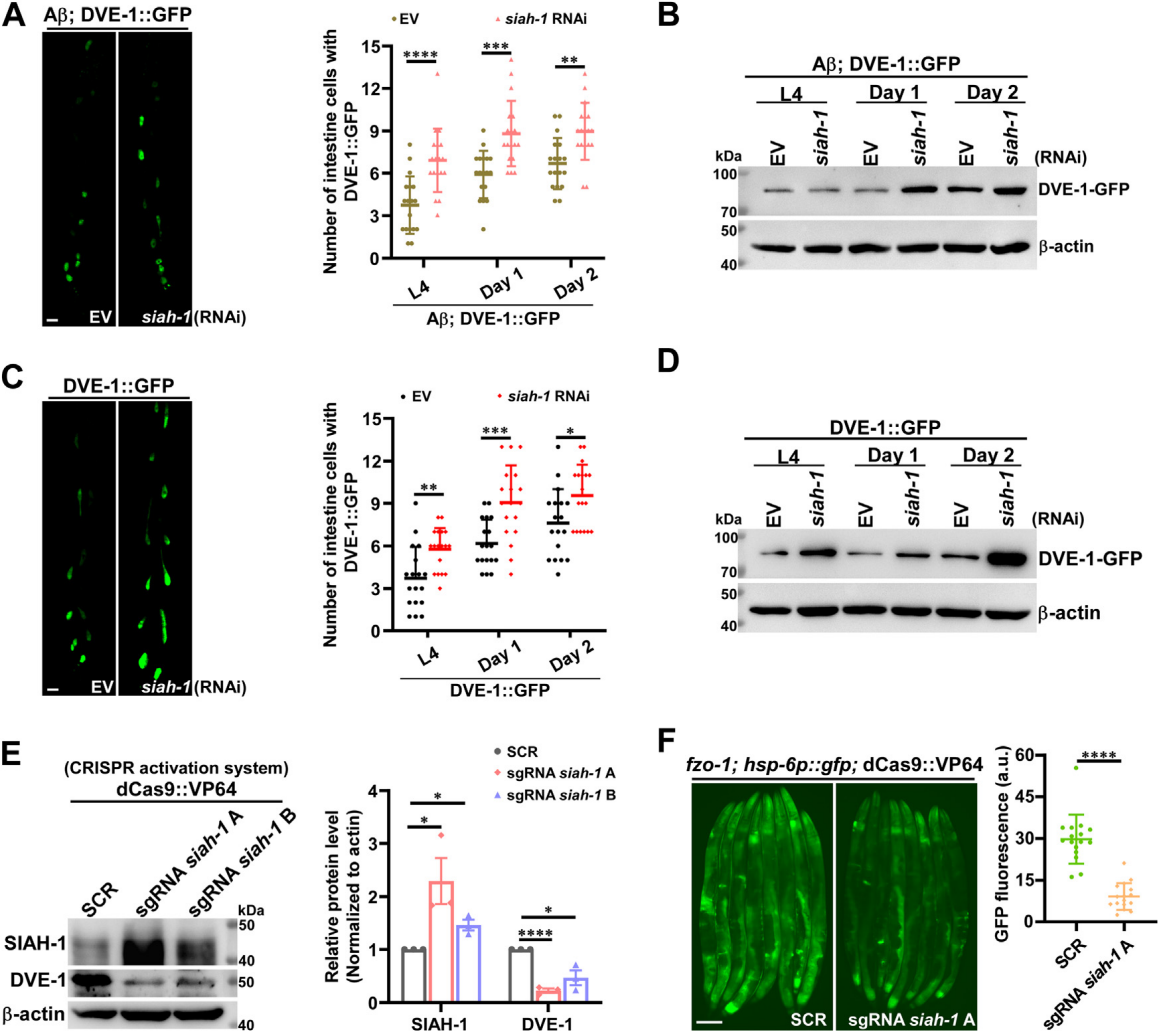
**SIAH-1 mediates DVE-1 degradation.***A*, fluorescence micrographs and quantification demonstrate DVE-1::GFP localization in intestinal nuclei of *dvIs2; zcIs39* worms treated with EV or *siah-1* RNAi. Representative images (scale bar: 5 μm) and analysis of GFP-positive nuclei per worm (n ≥ 15 per group, unpaired *t*-tests) show significantly increased *siah-1* in RNAi-treated animals compared to EV controls at different developmental stages (L4, day 1, day 2) (∗∗*p* < 0.01, ∗∗∗*p* < 0.001, ∗∗∗∗*p* < 0.0001). *B*, Western blot analysis of DVE-1::GFP and β-actin in *dvIs2; zcIs39* worms treated with EV or *siah-1* RNAi, harvested and examined at different developmental stages (L4, day 1, day 2). *C*, representative fluorescence images of DVE-1::GFP in intestinal nuclei of *zcIs39* worms treated with EV and *siah-1* RNAi. Scale bar: 5 μm. Quantification across L4, Day 1 and Day 2 stages showed more GFP-positive nuclei in *siah-1* RNAi-treated worms compared to EV controls (n ≥ 15 per group, unpaired *t*-tests; ∗*p* < 0.05, ∗∗*p* < 0.01, ∗∗∗*p* < 0.001). *D*, Western blot analysis of DVE-1::GFP and β-actin in *zcIs39* worms treated with EV and *siah-1* RNAi, collected and examined at different developmental stages (L4, Day 1 and Day 2). *E*, Western blot analysis of endogenous SIAH-1 and DVE-1 in *risIs33*(dCas9::VP64) worms fed with HT115 bacteria carrying sgRNA SCR, HT115 sgRNA *siah-1* A or sgRNA *siah-1* B. β-actin was used as a control. Normalized quantification revealed increased SIAH-1 expression and decreased DVE-1 level after treatment with sgRNA *siah-1* A or sgRNA *siah-1* B compared with SCR controls (three independent experiments, unpaired *t*-tests; ∗*p* < 0.05, ∗∗∗∗*p* < 0.0001). *F*, representative fluorescence micrographs of *fzo-1(tm1133); zcIs13(hsp-6p::gfp); risIs33(dCas9::VP64)* young adult worms fed with HT115 bacteria carrying sgRNA SCR or sgRNA *siah-1* A. Scale bar: 100 μm. Quantification showed a significant decrease in *hsp-6p::*GFP fluorescence after sgRNA *siah-1* A treatment compared to SCR controls (n ≥ 15 worms per group, unpaired *t* test; ∗∗∗∗*p* < 0.0001).

To investigate whether SIAH-1 mediates Aβ-associated DVE-1 downregulation, we treated Aβ; DVE-1::GFP worms with *siah-1* RNAi and confirmed SIAH-1 reduction by Western blot (Fig. S2*B*). While *dve-1* mRNA levels remained unaffected following *siah-1* RNAi treatment (Fig. S2*C*), we observed a significant increase in the number of intestinal cells expressing DVE-1::GFP in *siah-1* RNAi worms compared to control (empty vector, EV) RNAi animals at the same developmental stages (Fig. 2*A*). In addition, we examined total protein levels of DVE-1::GFP at different stages in Aβ; DVE-1::GFP worms treated with EV and *siah-1* RNAi and found that DVE-1::GFP was increased after *siah-1* knockdown (Fig. 2*B*). Similar results were obtained in DVE-1::GFP worms treated with *siah-1* RNAi, with a significantly higher number of intestinal cells expressing DVE-1::GFP (Fig. 2*C*) and an elevated total protein level of DVE-1::GFP compared to EV-treated animals (Fig. 2*D*). RNAi-mediated suppression of SIAH-1 was examined by Western blot (Fig. S2*D*). These results indicate that SIAH-1 regulates DVE-1 at the protein level.

We next tested whether *siah-1* overexpression accelerates DVE-1 degradation using the well-established CRISPR-activated (CRISPRa) expression system in *C. elegans* (31). Two sgRNA constructs targeting *siah-1* (sgRNA *siah-1* A and sgRNA *siah-1* B) were introduced into *risIs33*(dCas9::VP64) worms. qPCR and Western blot confirmed robust *siah-1* upregulation, particularly with sgRNA-*siah-1*-A (Figs. S2*E* and 2*E*). CRISPRa-mediated *siah-1* activation significantly reduced endogenous DVE-1 protein levels in both sgRNA *siah-1* A and B worms compared to scramble controls (SCR) (Fig. 2*E*), without decreasing *dve-1* mRNA (Fig. S2*F*). To assess functional consequences, we activated *siah-1* in *fzo-1* mutant worms containing both dCas9::VP64 and UPR^mt^ reporter (*hsp-6p::gfp*), where DVE-1 downregulation suppresses mitochondrial unfolded protein response (32). The activated UPR^mt^, as indicated by *hsp-6p::gfp* in *fzo-1(tm1133)* mutants*,* was significantly inhibited by sgRNA *siah-1* A compared to SCR treatment (Fig. 2*F*), indicating that endogenous activation of *siah-1* promotes the downregulation of DVE-1. Taken together, these results suggest that SIAH-1 mediates the degradation of DVE-1.

### E3 ligase activity of SIAH-1 is required for the proteasomal degradation of DVE-1

**Figure 3:**
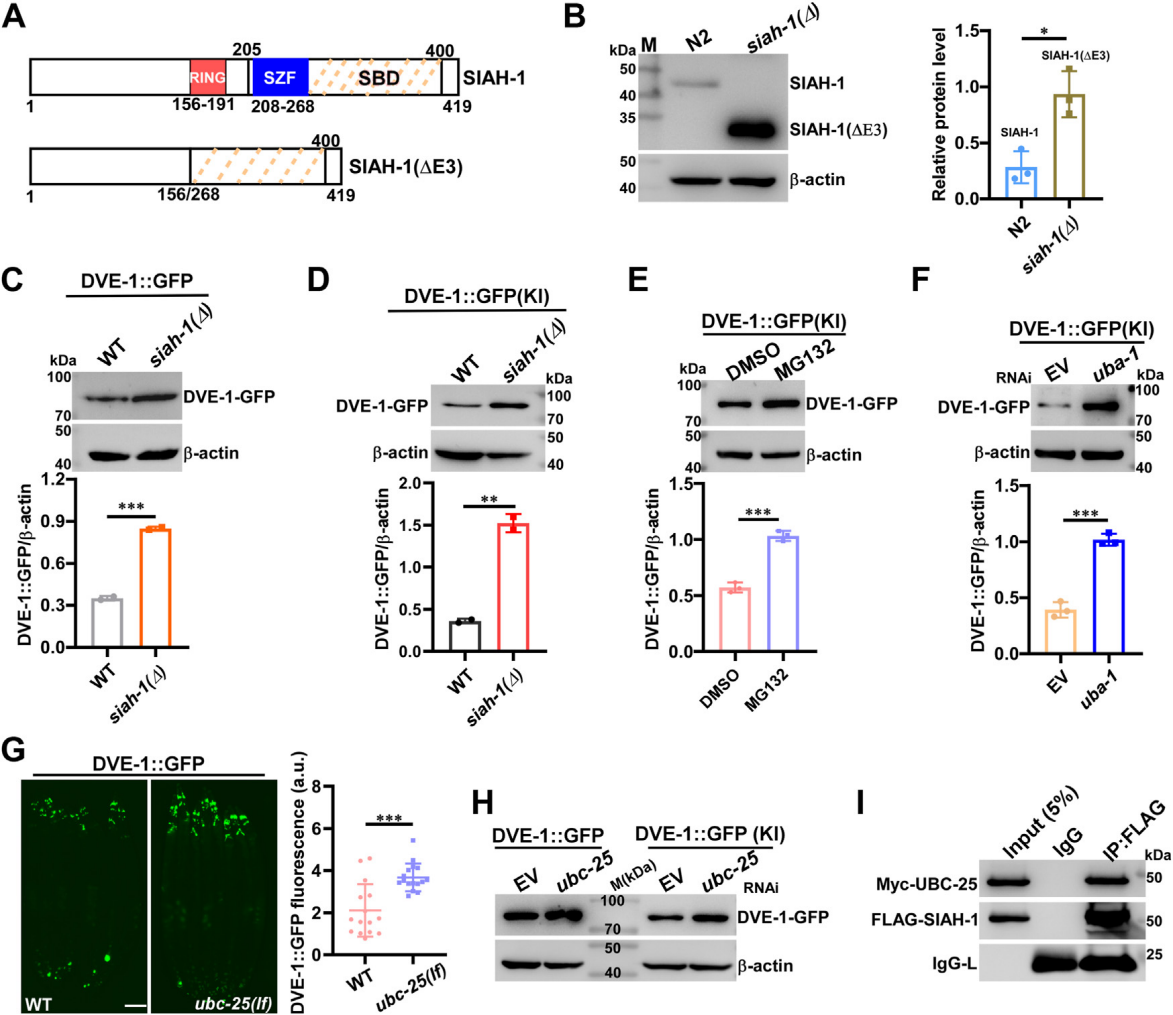
**SIAH-1 E3 ligase activity contributes to the proteasomal degradation of DVE-1.***A*, schematic representation of full-length SIAH-1 and truncated SIAH-1(ΔE3) based on UniProt database. Numbers indicate amino acid positions. RING domain (156-191 aa, red), Siah-type zinc finger motif (SZF, 208-268 aa, blue), and SBD (205-400 aa, dotted lines) are shown. *B*, Western blot analysis of SIAH-1 and SIAH-1(ΔE3) in N2 and *siah-1(syb4782)* mutants (denoted as *siah-1(Δ)*), respectively. Approximately 46 KD of full-length SIAH-1 was detected in N2, while approximately 33 KD of SIAH-1(ΔE3) was found in *siah-1(syb4782)* mutant worms. β-actin serves as a loading control. Quantification revealed increased SIAH-1(ΔE3) levels *versus* N2 (three experiments; unpaired *t*-tests; ∗*p* < 0.05). *C*, DVE-1::GFP and β-actin were examined in *zcIs39* and *syb4782; zcIs39* animals at day 1 stage. Quantification revealed that DVE-1::GFP was increased in *siah-1(syb4782)* mutants compared to WT controls (two experiments, ∗∗∗*p* < 0.001). *D*, DVE-1::GFP was examined in *syb4782; syb1984* animals using the DVE-1::GFP(KI) reporter. Quantification results similar to (*C*) (two independent experiments, ∗∗*p* < 0.01). *E*, Western blot analysis of DVE-1::GFP and β-actin protein levels in day 1 adult *syb1984* worms treated with DMSO or 10 μM MG132. Quantification revealed that DVE-1::GFP was increased after treatment with MG132 (three experiments; ∗∗∗*p* < 0.001). *F*, Western blot analysis of DVE-1::GFP and β-actin in *syb1984* worms treated with EV and *uba-1* RNAi. Quantification revealed increased DVE-1::GFP levels in *uba-1* RNAi compared to EV controls (three experiments; ∗∗∗*p* < 0.001). *G*, DVE-1::GFP fluorescence in wild-type and *ubc-25(ok1732)* worms. Scale bar: 100 μm. Quantification showed a significant increase in DVE-1::GFP levels in *ubc-25(ok1732)* mutants compared to WT controls (n ≥ 15 per group; ∗∗∗*p* < 0.001). *H*, Western blot analysis of DVE-1::GFP and β-actin in *zcIs39* and *syb1984* strains treated with EV or *ubc-25* RNAi, and DVE-1-GFP is increased after *ubc-25* RNAi. *I*, the interaction between UBC-25 and SIAH-1 was determined by Co-IP. HEK293 cells were transfected with the indicated expression plasmids of Myc-UBC-25 and FLAG-SIAH-1. Forty-eight hours after transfection, cell lysates were prepared and immunoprecipitated (IP) with anti-FLAG antibody or control IgG antibodies and immunoblotted with anti-Myc or anti-FLAG antibodies. IgG-L: light chains of anti-FLAG or normal IgG.

The *C. elegans* SIAH-1 belongs to the SIAH ubiquitin E3 ligase family, which is characterized by conserved functional domains (33). The N-terminal RING (Really Interesting New Gene) finger domain is essential for its E3 ubiquitin ligase activity, while the C-terminal substrate binding region, comprising SZF (SIAH-type zinc finger), SBD (substrate binding domain), and DIMER (dimerization) domains, facilitates substrate protein binding for degradation and homodimerization for auto-degradation (34, 35, 36, 37, 38). The domain architecture of *C. elegans* SIAH-1, based on UniProt database, is illustrated in Figure 3*A*.

**Figure S3:**
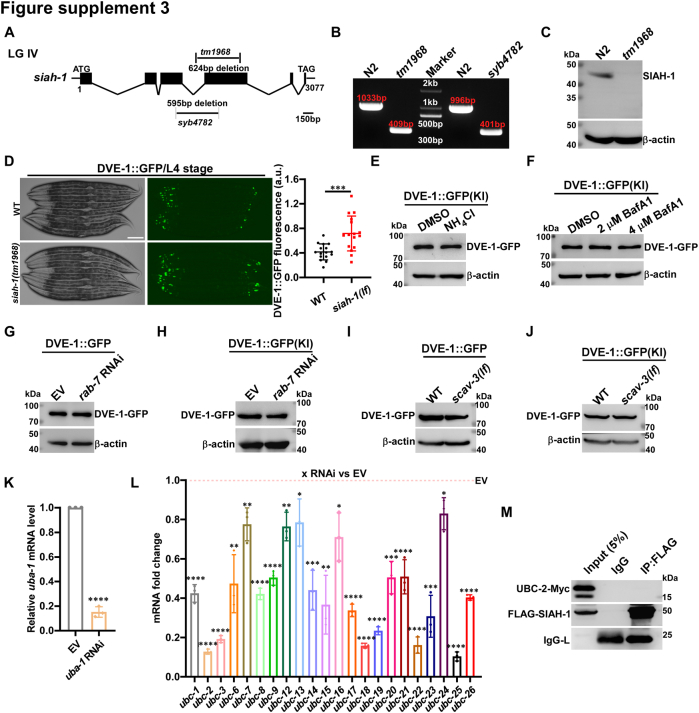


To verify the E3 ligase function of SIAH-1, we generated *siah-1(syb4782)* (*siah-1(Δ)*) mutant *via* CRISPR-Cas9 to delete 595 bp of genomic DNA encoding RING and SZF domains (Figs. S3*A* and 3*A*), confirmed by PCR genotyping (Fig. S3*B*). This in-frame deletion resulted in a smaller protein designated SIAH-1(ΔE3) (Fig. 3*A*). Western blot analysis using the SIAH-1 antibody detected full-length SIAH-1 (∼46 kDa) in N2 worms, whereas truncated SIAH-1(ΔE3) protein (∼33 kDa) was present in *siah-1(Δ)* animals (Fig. 3*B*, left panel). Notably, SIAH-1(ΔE3) accumulates significantly in *siah-1(Δ)* mutants, while wild-type SIAH-1 was relatively low in abundance in N2 worms (Fig. 3*B*), consistent with the model that the lack of E3 ubiquitin ligase activity in *siah-1(Δ)* prevents auto-ubiquitination and thereby its degradation.

To confirm that the E3 ligase activity of SIAH-1 is responsible for DVE-1 degradation, we introduced DVE-1::GFP and DVE-1::GFP(KI) reporters into the *siah-1(Δ)* mutants. Both reporters exhibited significantly increased levels of DVE-1-GFP in *siah-1(Δ)* mutants compared to wild-type animals (Fig. 3, *C* and *D*), consistent with observations in *siah-1(tm1968)* null allele mutant worms (Fig. S3, *B*–*D*).

We demonstrated that the levels of DVE-1-GFP were restored in Aβ; DVE-1::GFP worms after treatment with the proteasome inhibitor MG132 (Fig. 1*D*). To exclude the possible involvement of the lysosomal pathway in DVE-1 degradation, we treated DVE-1::GFP(KI) worms with the lysosomal inhibitors NH_4_Cl and bafilomycin A1 (39, 40, 41, 42). While MG132 treatment significantly increased DVE-1-GFP levels in DVE-1::GFP(KI) worms (Fig. 3*E*), treatment with NH_4_Cl or bafilomycin A1 showed no effect (Fig. S3, *E* and *F*). Furthermore, we examined DVE-1-GFP levels in DVE-1::GFP and DVE-1::GFP(KI) worms treated with *rab-7* RNAi as well as in *scav-3(ok1286)* mutant worms containing both reporters, as both *rab-7* RNAi and *scav-3(lf)* inhibit lysosomal activity through different mechanisms (43, 44, 45, 46, 47). Consistent with the results from NH_4_Cl and bafilomycin A1 treatment, neither *rab-7* RNAi nor *scav-3 (lf)* elevated DVE-1-GFP levels (Fig. S3, *G*–*J*), indicating that the lysosomal pathway is not responsible for DVE-1 turnover.

Collectively, these data suggest that SIAH-1 promotes proteasomal degradation of DVE-1 *via* its E3 ligase activity.

### SIAH-1 conjugates with UBC-25 to facilitate degradation of DVE-1

To further validate that DVE-1 is degraded *via* the UPS pathway, we tested whether knockdown of genes related to the ubiquitin-activating enzyme (E1) and ubiquitin-conjugating enzyme (E2) would enhance DVE-1 expression. In *C. elegans*, disruption of the sole E1 enzyme gene, *uba-1*, has been shown to result in complete inactivation of the UPS proteolysis (48, 49). We performed *uba-1* RNAi in DVE-1::GFP(KI) worms, confirmed *uba-1* mRNA reduction by qPCR (Fig. S3*K*), and observed a significant increase in DVE-1-GFP expression compared to EV RNAi controls (Fig. 3*F*).

*C. elegans* possesses 22 E2 enzymes (UBC-1-3, UBC-6-9, and UBC-12-26) (50, 51). To identify which E2 enzyme partners with SIAH-1 to target DVE-1, we performed RNAi of UBC-related genes in DVE-1::GFP animals, confirming RNAi efficacy by qPCR (Fig. S3*L*). We observed that knockdown of *ubc-25* significantly increased the fluorescence of DVE-1-GFP (Table S1), a result also observed in *ubc-25(ok1732)* mutant worms (Fig. 3*G*). This phenotype was also verified by Western blot after *ubc-25* RNAi treatment (Fig. 3*H*), confirming that disruption of *ubc-25* promotes DVE-1::GFP expression.

Since E3 ligases typically pair with E2 enzymes to facilitate the degradation of substrate proteins (52), we examined the interaction between SIAH-1 and UBC-25 using Co-Immunoprecipitation (Co-IP) assays. Our results demonstrated physical interaction between SIAH-1 and UBC-25 (Fig. 3*I*). While *ubc-2* RNAi also resulted in increased expression of DVE-1-GFP (Table S1), we detected no interaction between UBC-2 and SIAH-1 (Fig. S3*M*), suggesting UBC-25 specifically partners with SIAH-1 for targeting DVE-1.

### SIAH-1 physically interacts with DVE-1

**Figure 4:**
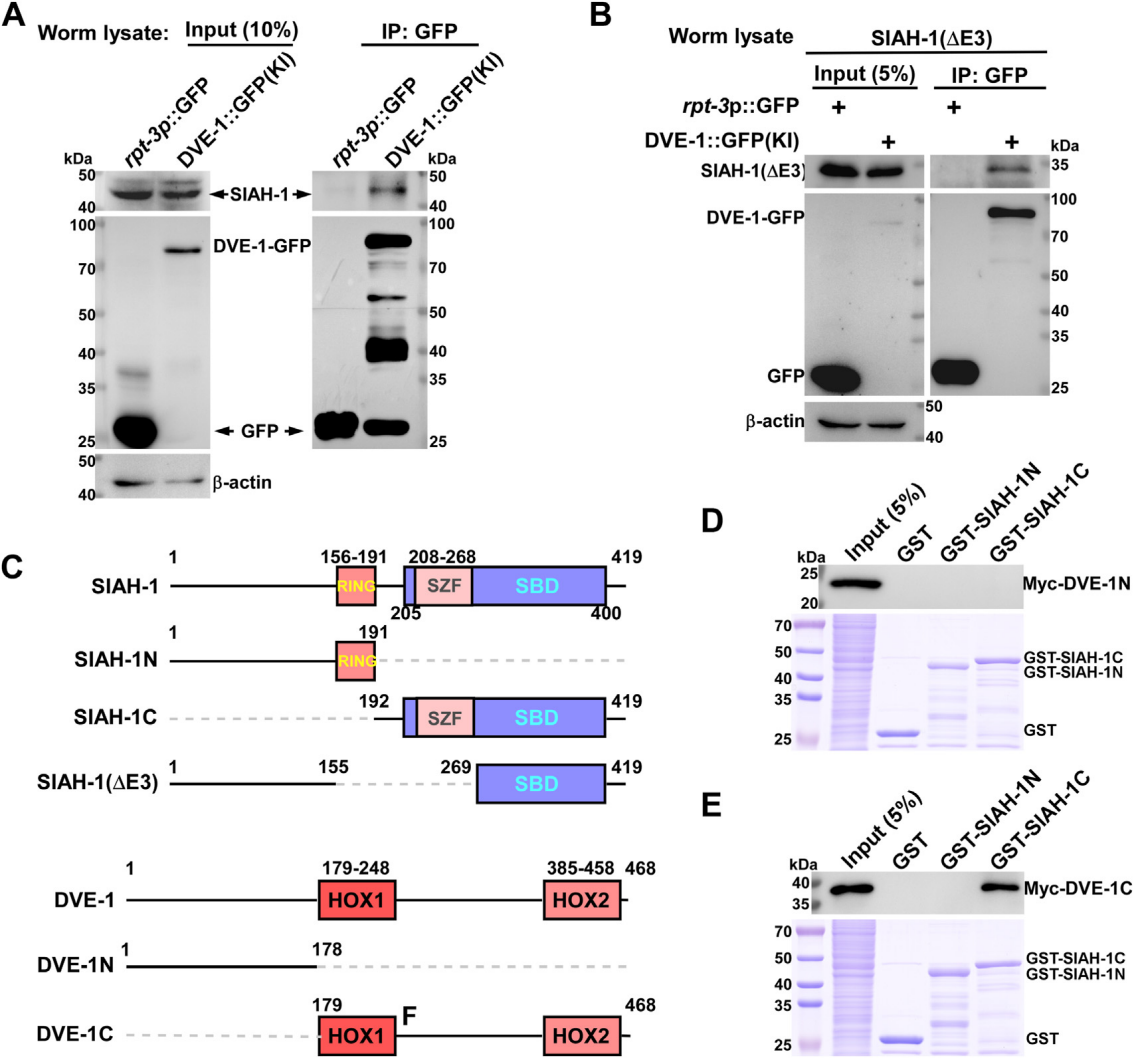
**SIAH-1 physically interacts with DVE-1.***A*, Co-IP analysis of SIAH-1 and DVE-1 interaction in *mgIs72(rpt-3p::gfp)* and DVE-1::GFP(KI) (*syb1984)* worms. *mgIs72* serves as a negative control. Worm lysates were immunoprecipitated with anti-GFP nanobody. Pellets were probed by Western blot using anti-GFP and anti-SIAH-1 antibodies. *B*, Co-IP analysis of SIAH-1 (ΔE3) and DVE-1 interactions in *siah-1(syb4782); mgIs72(rpt-3p::gfp)* and *syb4782; syb1984(dve-1::gfp)* worms. lysates were immunoprecipitated with anti-GFP nanobody and probed with anti-GFP and anti-SIAH-1 antibodies. *C*, schematic representation of full-length SIAH-1, DVE-1 and truncated SIAH-1, DVE-1 proteins. Numbers indicate the amino acid positions. RING domain, zinc finger motif, and SBD region are shown in color. DVE-1 N-terminus (1-178 aa) and C-terminus (179-468 aa) are indicated. *D*, GST pull-down assay using purified GST, GST-SIAH-1(1-191), or GST-SIAH-1(192-419) incubated with cell lysates expressing Myc-tagged DVE-1(1-178). Bound proteins were analyzed by Western blot using anti-Myc antibodies. *E*, GST pull-down assay using purified GST, GST-SIAH-1(1-191), or GST-SIAH-1(192-419) incubated with cell lysates expressing Myc-tagged DVE-1(179-468), and then bound proteins were analyzed by Western blotting using anti-Myc antibodies.

Next, we investigated whether SIAH-1 interacts with DVE-1. IP assays were performed using anti-GFP nanobodies in *rpt-3p::*GFP, and DVE-1::GFP(KI) worms (Fig. 4*A*), as well as in their *siah-1(Δ)* mutant counterparts (Fig. 4*B*). Our results demonstrated that both full-length SIAH-1 and the truncated SIAH-1(ΔE3) interact with DVE-1 (Fig. 4, *A* and *B*), confirming a stable interaction independent of SIAH-1’s E3 ligase activity.

**Figure S4:**
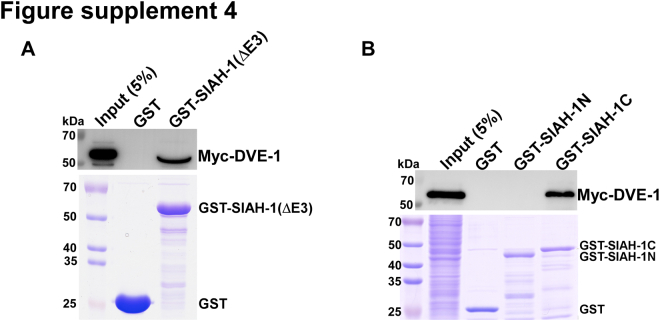


To map the interaction regions of SIAH-1 and DVE-1, we constructed truncated mutants fused to GST and Myc tags, respectively (Fig. 4*C*). Through GST pulldown assays, we observed that both SIAH-1(ΔE3) and SIAH-1C (192-419 aa) interacted with DVE-1 (Fig. S4, *A* and *B*), indicating that the RING and SZF domains are dispensable for DVE-1 binding. Instead, SIAH-1 interacts with DVE-1 through its SBD region (269-419 aa) (Fig. S4*B*). Furthermore, we found that the C-terminal region of DVE-1(DVE-1C, 179-468 aa) but not the N-terminal region (DVE-1N, 1-178 aa) binds to SIAH-1C (192-419 aa) (Fig. 4, *D* and *E*).

Taken together, these data suggest that SIAH-1, like other RING-type E3 ligases, binds its target DVE-1 *via* the SBD region, specifically interacting with the C-terminus of DVE-1.

### SIAH-1 and UBC-25 promote the K48-linked ubiquitination of DVE-1

**Figure 5:**
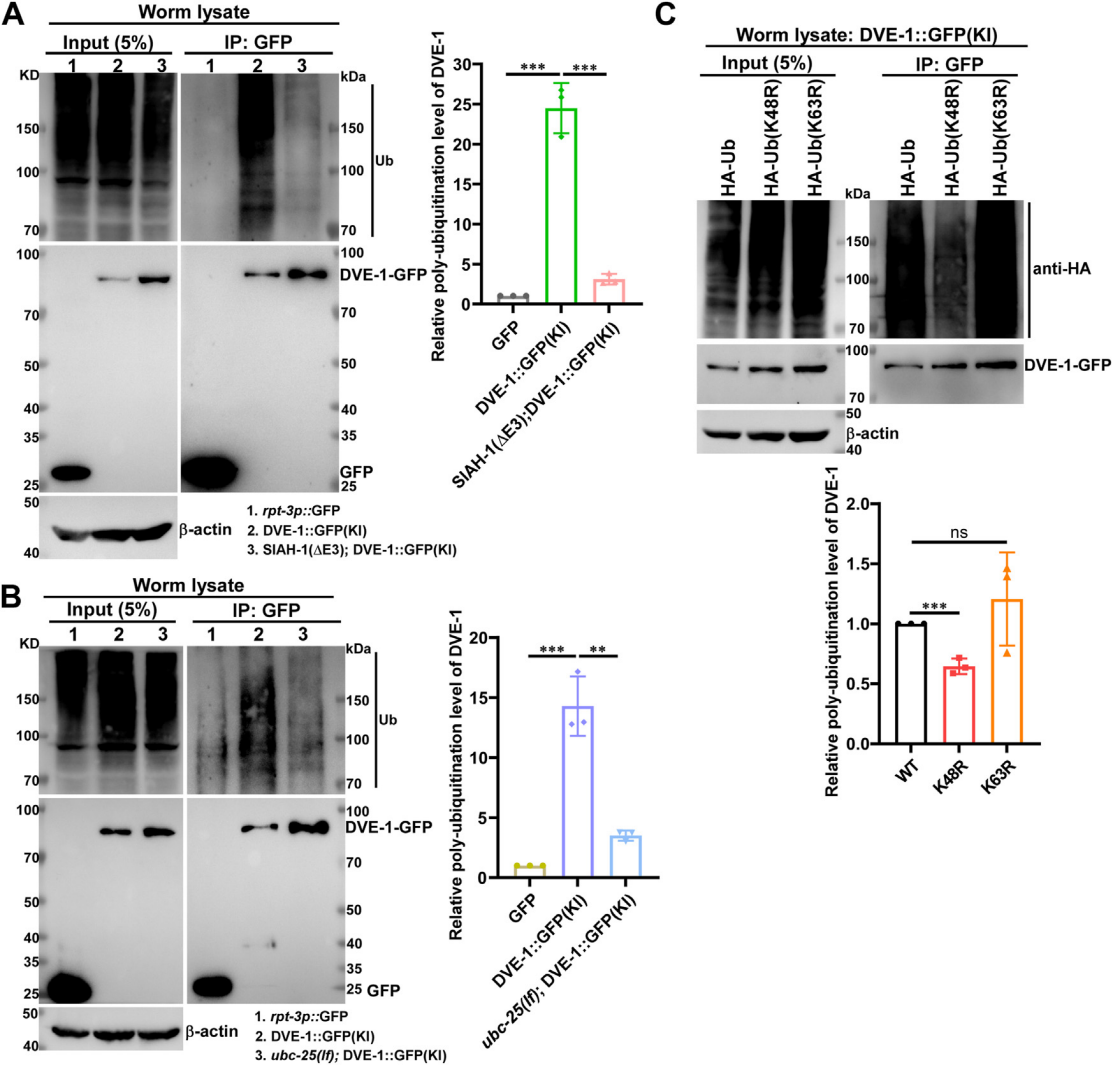
**SIAH-1 and UBC-25 promote DVE-1 ubiquitination.***A*, ubiquitination of DVE-1::GFP was examined in *mgIs72(rpt-3p::gfp)* (lane 1), *syb1984(dve-1::gfp)* (lane 2), and *siah-1(syb4782); syb1984* worms (lane 3). The *mgIs72* strain served as a negative control. Co-IP was performed using anti-GFP nanobody, followed by immunoblotting with anti-ubiquitin and anti-GFP antibodies. DVE-1 ubiquitination levels were quantified using ImageJ software. The ratio of ubiquitin to DVE-1::GFP was determined and normalized to the *mgIs72* control group (three experiments; unpaired *t*-tests; ∗∗∗*p* < 0.001). *B*, DVE-1 ubiquitination was analyzed in *mgIs72(rpt-3p::gfp)* (lane 1), *syb1984(dve-1::gfp)* (lane 2), and *ubc-25(ok1732); syb1984* worms (lane 3). Co-IP and immunoblotting were performed as described in (*A*). Quantification of ubiquitinated DVE-1 levels (normalized to DVE-1::GFP) was performed using ImageJ software and expressed relative to the *mgIs72* control group (three experiments; ∗∗*p* < 0.01, ∗∗∗*p* < 0.001). *C*, ubiquitination of DVE-1 was examined in worms carrying HA::UBQ-2(WT); DVE-1::GFP(KI), HA::UBQ-2(K48R); DVE-1::GFP(KI) or HA::UBQ-2(K63R); DVE-1::GFP(KI). Co-IP was performed using anti-GFP nanobody, followed by immunoblotting with anti-HA and anti-GFP antibodies. Ubiquitinated DVE-1 levels (normalized to DVE-1::GFP) were quantified with ImageJ software and expressed relative to the WT ubiquitination level (three experiments; ∗∗∗*p* < 0.001, ns, not significant).

To further elucidate that SIAH-1 functions as an E3 ligase to mediate DVE-1 degradation *via* the UPS pathway, we investigated the effect of SIAH-1(ΔE3) on DVE-1 polyubiquitination. IP assays were conducted using anti-GFP nanobodies in *rpt-3p*::GFP (negative control), DVE-1::GFP(KI), and *siah-1(Δ);* DVE-1::GFP(KI) worms. DVE-1 was notably poly-ubiquitinated in DVE-1::GFP(KI) worms (Fig. 5*A*, lane 2), whereas this polyubiquitination was significantly reduced in *siah-1(Δ)* mutants (Fig. 5*A*, lane 3). Consistently, *ubc-25(lf)* mutants also exhibited markedly decreased DVE-1 polyubiquitination compared to wild-type worms (Fig. 5*B*), indicating that both E3 ligase SIAH-1 and E2 enzyme UBC-25 mediate DVE-1 poly-ubiquitination.

K48- and K63-linked ubiquitination are the most predominant forms of polyubiquitination (53). K48-linked polyubiquitin chains primarily target proteins for 26S proteasomal degradation (54), while polyubiquitin formed by K63 conjugation is involved in the regulation of endocytosis or changes in target protein function (55, 56). To determine the specific type of ubiquitin linkage, we introduced DVE-1::GFP(KI) into *xwh20*(HA::Ub), *xwh23*(HA::Ub K48R), or *xwh24*(HA::Ub K63R) strains. DVE-1 polyubiquitination showed no significant difference between WT Ub and Ub (K63R) (Fig. 5*C*), while K48R ubiquitin significantly abolished DVE-1 polyubiquitination (Fig. 5*C*).

Collectively, these results demonstrated that SIAH-1 and UBC-25 catalyze K48-linked polyubiquitination of DVE-1, targeting it for proteasomal degradation.

### Loss of SIAH-1 E3 ligase activity or overexpression of DVE-1 mitigates A**β** toxicity

**Figure 6:**
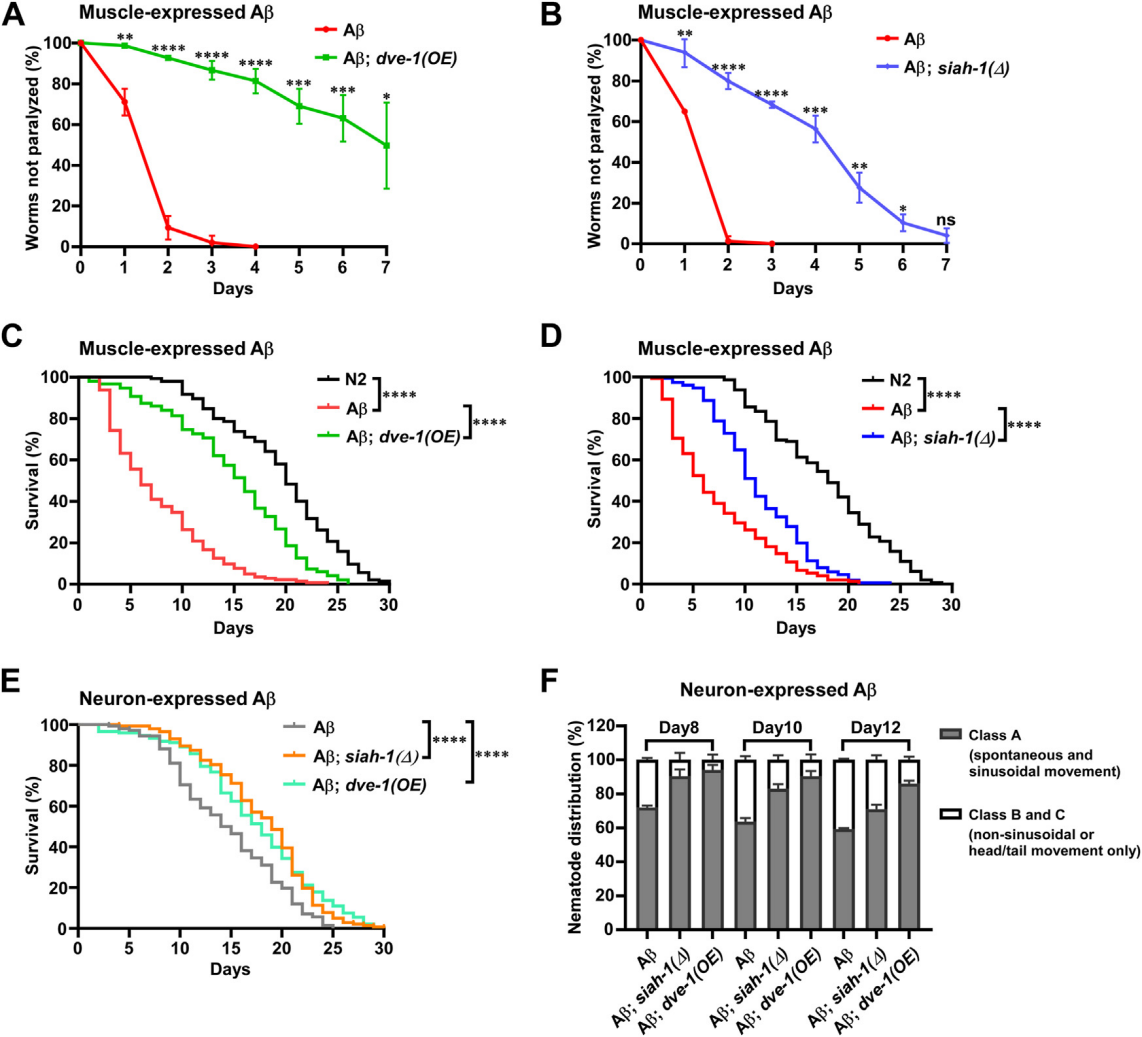
**Loss of SIAH-1 E3 ligase or overexpression of DVE-1 mitigates Aβ toxicity.***A*, assessment of paralysis in *dvIs2 (unc-54p::*Aβ_1-42_*)* and *dvIs2; zcIs39(dve-1::gfp)* worms after transfer to 25 °C. Data represent the mean ± SD from three independent experiments (n = 50 per experiment). Comparison at each time point was made using unpaired *t*-tests (∗*p* < 0.05, ∗∗*p* < 0.01, ∗∗∗*p* < 0.001, ∗∗∗∗*p* < 0.0001). *B*, paralysis analysis in *dvIs2* and *dvIs2; siah-1(syb4782)* worms after transfer to 25 °C. Data show the mean ± SD of three independent experiments (n = 50 per experiment). Statistical comparisons at each time point were performed using unpaired *t*-tests (∗*p* < 0.05, ∗∗∗*p* < 0.001, ∗∗∗∗*p* < 0.0001, ns:not significant). *C*, survival analysis of wild-type N2, *dvIs2* and *dvIs2; zcIs39* worms. See Table S2 for survival statistics (∗∗∗∗*p* < 0.0001, log-rank test). *D*, lifespan analysis of wild type N2, *dvIs2* and *dvIs2; siah-1(syb4782)* worms. Detailed lifespan statistics are included in Table S2 (∗∗∗∗*p* < 0.0001; log-rank test). *E*, lifespan analysis of *gnaIs2(unc-119p::*Aβ_1-42_*)* and *gnaIs2; siah-1(syb4782) and gnaIs2; zcIs39* worms. Statistical details are provided in Table S2 (∗∗∗∗*p* < 0.0001; log-rank test). *F*, the Percentage of Class A nematodes was significantly increased in *gnaIs2; siah-1(syb4782)* and *gnaIs2; zcIs39* worms compared to *gnaIs2* controls (n > 90; two-way ANOVA, Post hoc Tukey’s test; ∗∗∗∗*p* < 0.0001).

Our finding that SIAH-1 promotes UPS-mediated DVE-1 degradation in Aβ-expressing worms (Fig. S5*A*) prompted investigation of the therapeutic potential. In the muscle-specific Aβ model (CL2006), worms exhibited complete paralysis within 3 to 4 days at 25 °C (Fig. 6, *A* and *B*). Strikingly, either *dve-1(OE)* or *siah-1(Δ)* mutations significantly delayed paralysis compared to Aβ controls (Fig. 6, *A* and *B*). Notably, Aβ; *siah-1(Δ); dve-1(OE)* worms did not provide additional protective effects compared to Aβ; *siah-1(Δ)* animals (Fig. S5*B*), suggesting these interventions act through a shared mechanism. Furthermore, lifespan analysis indicated that Aβ worms displayed a shortened lifespan compared to wild-type N2 animals (Fig. 6, *C* and *D*, Table S2), whereas *dve-1(OE)* or *siah-1(Δ)* substantially extended the lifespan of Aβ-expressing worms (Fig. 6, *C* and *D*, Table S2).

To assess nervous system applicability, we then analyzed *siah-1(Δ) or dve-1(OE)* in *gnaIs2*, a neuronal Aβ model expressing Aβ_1-42_ under the pan-neuronal *unc-119* promoter (57). Consistent with observations in the muscle-specific Aβ model (CL2006), both *siah-1(Δ)* and *dve-1(OE)* significantly extended lifespan (Fig. 6*E* and Table S2) and markedly improved motility in worms expressing neuronal Aβ (Fig. 6*F*), confirming the neuroprotective efficacy of these genetic interventions in mitigating Aβ-associated toxicity.

### Inactivation of SIAH-1 E3 ligase or overexpression of DVE-1 improves mitochondrial homeostasis to inhibit A**β** aggregates

**Figure 7:**
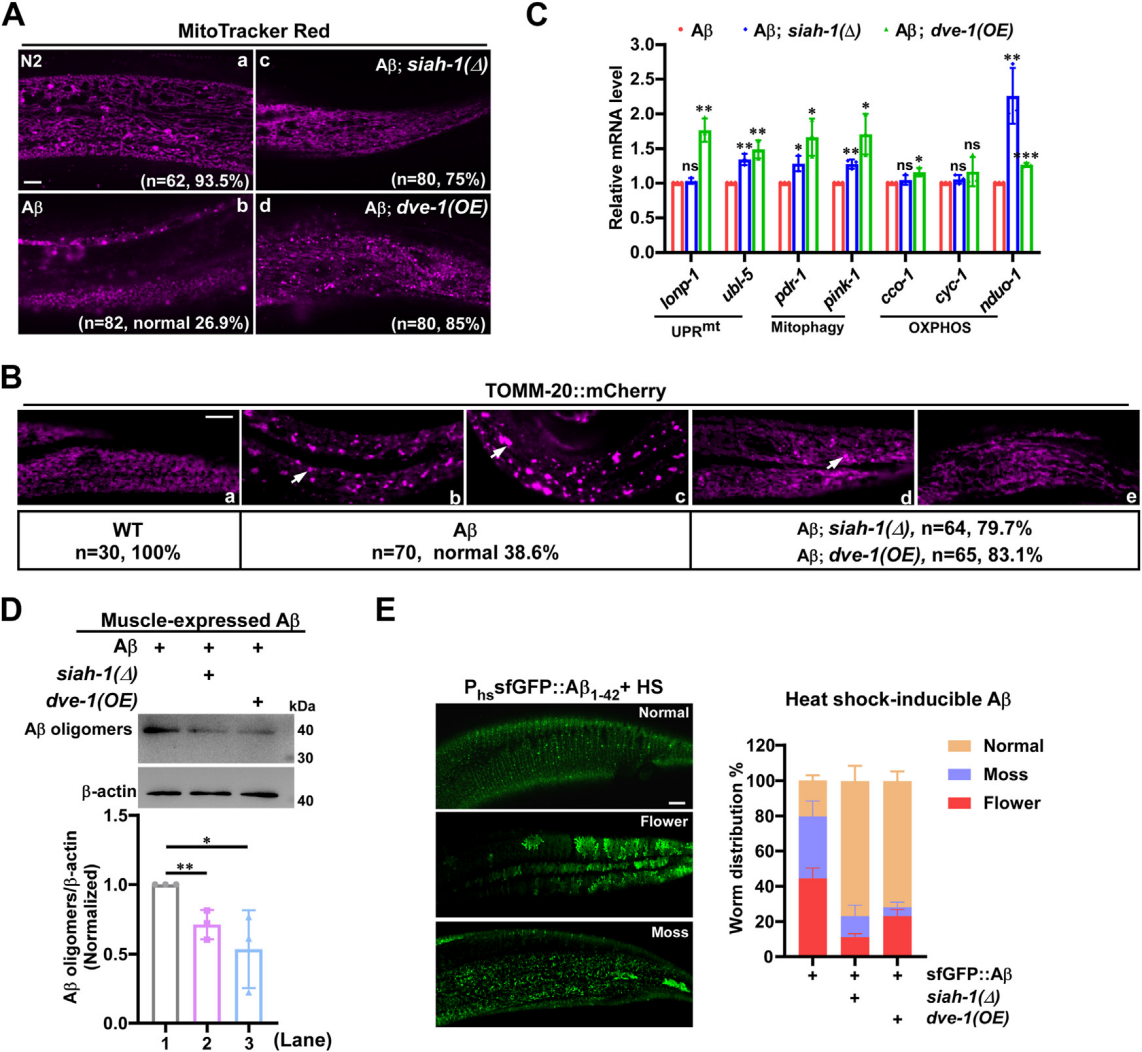
**Loss of SIAH-1 E3 ligase or overexpression of DVE-1 inhibits Aβ aggregation by improving mitochondrial homeostasis.***A*, representative confocal fluorescence images of mitochondria in wild-type N2, *dvIs2(unc-54p::*Aβ_1-42_*)*, *dvIs2; siah-1(syb4782)* and *dvIs2; zcIs39* worms with Mito Tracker Red. Images were captured on day 1 after transfer to 25 °C. Scale bar: 10 μm. *B*, hypodermal mitochondria labeled with TOMM-20::mCherry in wild-type, *dvIs2*, *dvIs2; siah-1(syb4782)*, and *dvIs2; zcIs39* animals. Arrows indicate TOMM-20::mCherry aggregates. At least 30 worms per genotype were analyzed. Scale bar: 10 μm. *C*, relative mRNA expression levels of *lonp-1*, *ubl-5, pdr-1, pink-1, cco-1, cyc-1* and *nduo-1* in *dvIs2*, *dvIs2; siah-1(syb4782)* and *dvIs2; zcIs39* worms on day 1 after transfer to 25 °C, as determined by qPCR. Data are normalized to the *dvIs2* group (three experiments; unpaired *t*-tests; ∗*p* < 0.05, ∗∗*p* < 0.01, ∗∗∗*p* < 0.001, ns, not significant). *D*, Western blot analysis of Aβ oligomers in *dvIs2, dvIs2; siah-1(syb4782)* and *dvIs2; zcIs39* worms on day 1 after transfer to 25 °C. β-actin was used as a loading control. Aβ oligomer levels (normalized to β-actin) were quantified using ImageJ and expressed relative to the *dvIs2* group (three experiments; ∗*p* < 0.05, ∗∗*p* < 0.01). *E*, representative confocal fluorescence micrographs of sfGFP:: Aβ in *xchIs15* transgenic worms following heat-shock at 33 °C for 2 h. Images of the posterior hypodermis were captured after a 24-h recovery period at 20 °C. Moss-like and flower-like morphologies denote distinct Aβ aggregates. The absence of these structures in the posterior hypodermis was considered normal. Scale bar: 10 μm. Quantification of normal, moss-like and flower-like sfGFP:: Aβ morphologies in *xchIs15, siah-1(syb4782); xchIs15* and *zcIs39; xchIs15* worms. Data represent mean ± SD from three independent experiments (n = 118 for *xchIs15*; n = 83 for *siah-1(syb4782); xchIs15*; n = 89 for *zcIs39; xchIs15*; two-way ANOVA, Post hoc Tukey’s test; ∗∗∗∗*p* < 0.0001).

Given the critical role of DVE-1 in UPR^mt^ and the maintenance of mitochondrial morphology (11, 12, 58), we next sought to determine whether *siah-1(Δ)* or *dve-1(OE)* preserves mitochondrial integrity under Aβ stress. MitoTracker staining revealed that mitochondria in Aβ paralyzed worms were more fragmented and less connected, with a close-to-normal morphology observed in only 26.9% of cases (n = 82), compared to 93.5% in wild-type N2 animals (n = 61) (Fig. 7, *A*–*a*, *b*). In contrast, mitochondrial morphology was strongly restored in *siah-1(Δ)* mutants (75%, n = 80; Fig. 7, *A*–c) or *dve-1*(OE) (85%, n = 80; Fig. 7, *A*–d). To confirm these results, we used TOMM-20::mCherry, a mitochondrial outer membrane marker (59). While WT worms showed uniform TOMM-20::mCherry distribution (100% normal, n = 30; Fig. 7, *B*–a), Aβ worms displayed pathological morphological alterations, characterized by numerous TOMM-20::mCherry aggregates exhibiting varying sizes (38.6% normal, n = 70; Fig. 7, *B*–*b*, *c*) (Fig. 7, *B*–*b*, *c*). Similar to the results of MitoTracking staining, both *siah-1(Δ)* (79.7%, n = 64) and *dve-1(OE)* (83.1%, n = 65) greatly reduced TOMM-20::mCherry aggregates and rescued the mitochondrial abnormal morphology (Fig. 7, *B*–*d*, e). In addition, transcript analysis of genes involved in mitochondrial stress responses, including the *lonp-1, ubl-5*, *pdr-1*, *pink-1*, *cco-1*, *cyc-1* and *nduo-1*, revealed that most of these genes were upregulated in both Aβ; *siah-1(Δ)* and Aβ; *dve-1(OE)* worms compared to Aβ worms alone (Fig. 7*C*), supporting improved mitochondrial homeostasis.

Previous study showed that Aβ oligomers but not high-molecular-weight aggregates correlate with toxicity (60). Since mitochondrial homeostasis has been reported to counteract Aβ proteotoxicity (5), we then assessed Aβ aggregation in Aβ; *siah-1(Δ)* and Aβ; *dve-1(OE)* worms by Western blot after 24h at 25°C. Aβ aggregates (∼40 kD) were significantly reduced in both Aβ; *siah-1(Δ)* and Aβ; *dve-1(OE)* worms compared to Aβ worms (Fig. 7*D*), indicating that *siah-1(Δ)* or *dve-1*(OE) significantly inhibited Aβ aggregation. To confirm these findings, we used another *C. elegans* AD transgenic strain, *xchIs15(hsp-16.2p*::ss::sfGFP::Aβ_1-42_*)*, which expresses and secretes sfGFP::Aβ to form aggregates with “flower” and “moss” patterns in the extracellular space after heat shock induction (61). Consistent with the results in muscle-specific Aβ expression worms, both the “flower” and the “moss” patterns of sfGFP::Aβ aggregates in the hypodermis were significantly reduced in *siah-1(Δ)* or *dve-1* (OE) animals compared to control worms (Fig. 7*E*).

Taken together, these data suggest that loss of SIAH-1 or overexpression of DVE-1 reduces Aβ aggregation to alleviate Aβ toxicity by enhancing mitochondrial homeostasis.

## Discussion

There is currently no cure for AD, the most common form of dementia in the elderly. Previous studies have shown that the UPR^mt^ is activated in patients with mild AD and in Aβ-related animal models (5, 6, 7, 8). Understanding the intracellular relationship between Aβ and UPR^mt^ factors may contribute to AD prevention and the development of novel therapeutic strategies. In this study, using the *C. elegans* muscle-expressed Aβ model, we discovered that DVE-1, a key TF in the UPR^mt^ signaling pathway, is degraded *via* the UPS due to the upregulation of the E3 ligase SIAH-1 in the presence of Aβ. Loss of SIAH-1 or overexpression of DVE-1 significantly reduces Aβ aggregation and attenuates Aβ toxicity, suggesting a potential therapeutic approach for conditions characterized by Aβ proteotoxicity.

The UPR^mt^ alleviates mitochondrial stress and restores mitochondrial proteostasis by inducing mitochondrial chaperones and proteases, as well as by rewiring cellular metabolism, a process well characterized in *C. elegans* (58, 62). The turnover of DVE-1, a key regulator of UPR^mt^ signaling, remains unexplored. Here, we identified SIAH-1 as a specific E3 ligase for DVE-1. In RING domain-deleted mutants of SIAH-1 or in *siah-1* RNAi worms, DVE-1 protein accumulated to excessive levels (Figs. 2, *A*–*D* and 3, *C* and *D*), whereas overexpression of *siah-1* reduced DVE-1 expression (Fig. 2*E*), thereby alleviating cell non-autonomous UPR^mt^ in *fzo-1(tm1133)* mutant animals (Fig. 2*F*). Furthermore, SIAH-1 interacts with the E2 conjugation enzyme UBC-25 to facilitate the polyubiquitination of DVE-1 (Figs. 3*I* and 5, *A* and *B*), with the SBD domain of SIAH-1 physically targeting the C-terminus of DVE-1 for proteasomal degradation (Fig. 4).

The E2 enzyme UBC-25 and its human homologue UBE2QL1 play essential roles in cellular homeostasis, particularly in neuromuscular function and lysosomal integrity (63, 64). In addition, *ubc-25* functions redundantly with *scav-3* to maintain lysosomal integrity (63). However, unlike *ubc-25(lf)*, the *scav-3* mutation did not result in increased DVE-1 levels (Figs. 3, *G* and *H* and S3, *I* and *J*), indicating that the lysosomes are not responsible for DVE-1 degradation. This highlights the specific role of UBC-25 as an E2 enzyme in regulating DVE-1 turnover. Homozygous *unc-25(ok1732); dvIs2(unc-54p::*Aβ*)* worms were not viable. Due to its essential role in lysosomal function, *ubc-25* RNAi in *dvIs2* worms exhibited increased paralysis compared to EV treatment, which could be significantly ameliorated by *siah-1(Δ)* (Fig. S5*C*) or *dve-1*(OE) (Fig. S5*D*), highlighting the role of mitochondrial homeostasis in counteracting Aβ toxicity.

*C. elegans* SIAH-1 is an evolutionarily conserved RING-finger type E3 ubiquitin ligase, characterized by the presence of eight cysteines in the RING domain that are critical for its E3 activity. In mammals, SIAH1 targets various proteins, including itself, for ubiquitylation and proteasomal degradation, participating in various biological processes and implicated in diseases such as cancer (65, 66, 67). Recent studies have demonstrated that SIAH1 undergoes K29-linked auto-ubiquitination and facilitates K27-linked ubiquitination of USP19 for proteasomal degradation (29). Our research revealed that SIAH-1 primarily promotes K48-linked ubiquitination of DVE-1 (Fig. 5), although other types of ubiquitination are not completely excluded. While *dve-1(OE)* outperformed *siah-1(Δ)* in mitigating Aβ toxicity, the combined *siah-1(Δ); dve-1(OE)* phenotype resembled *siah-1(Δ)* alone (Fig. S4*B*), suggesting additional SIAH-1 targets in this pathway.

Intriguingly, SIAH1 has been detected in the Lewy bodies of patients with PD, suggesting its involvement in the pathogenesis of the age-related neurodegenerative disease (21). Our studies indicate that the transcriptional expression of *siah-1* is upregulated in the presence of Aβ, and loss of SIAH-1 E3 ligase activity restoring DVE-1 expression and enhances mitochondrial proteostasis, thereby significantly reducing Aβ toxicity. However, the mechanism linking Aβ to *siah-1* induction and potential co-targets remain unclear. Notably, *siah-1(Δ)* or *dve-1(OE)* alleviated Tau-induced pathologies, including shortened lifespan and motor deficits (Fig. S5, *E* and *F*). Together, these findings position SIAH-1 as a potential therapeutic target for protein aggregation-related diseases, such as PD and AD.

## Experimental procedures

### *C. elegans* strains and genetics

All *C. elegans* strains were cultured at 20 °C on standard NGM plates seeded with *Escherichia coli* OP50, except for Aβ-related transgenic lines, which were maintained at 15 °C. The Bristol strain N2 was used as the wild type. Deletion mutants and transgenic strains were outcrossed with the N2 strain at least four times before phenotypic analysis. *C. elegans* culture maintenance and genetic crosses were performed following standard procedures described in the WormBook. The strains used in this study are listed in Table S3.

### RNAi treatment

For RNAi targeting *siah-1*(1260 bp), *ubc-3*(732 bp), *ubc-13*(456 bp), and *ubc-14*(*513 bp*), full-length coding sequences were cloned into pPD129.36 vector using standard molecular biology techniques. The vector was digested with HindIII and XbaI restriction enzymes, and the inserts were incorporated using Gibson assembly. Primer sequences are provided in Table S4. Additional RNAi clones were sourced from the Ahringer Genomic RNAi Library and verified by sequencing before use. RNAi efficiency of each gene was validated by qPCR (qPCR Primers are shown in Table S4). Feeding RNAi was performed as previously described (45). Briefly, adult worms were bleached and grown from hatch on *E. coli* HT115 strains expressing double-stranded RNA or harboring an empty vector control. Synchronized L4-stage worms were subsequently transferred to fresh RNAi plate seeded with the corresponding RNAi bacteria for further incubation and phenotypic analysis.

### RNA isolation and quantitative PCR analyses

Synchronized worms were collected from the plates and washed extensively with M9 buffer, worm pellets were resuspended in TRIzol reagent (Invitrogen). Samples were frozen in liquid nitrogen and thawed three times to crack worms. Total RNA was isolated by phenol-chloroform extraction, followed by isopropanol precipitation and DNase treatment. In addition to traditional RNA extraction methods, a simple method using proteinase K and a rapid heat treatment to release RNA (68) was also performed to determine RNAi efficiency. The cDNA was then synthesized using the M-MLV reverse transcriptase (Invitrogen). Gene expression levels were determined by quantitative real-time PCR using SYBR GREEN PCR Master Mix (AG11701) and Applied Biosystems 7500 system. Quantification of transcripts was normalized to *rpl-32* or *Y45F10D.4* mRNA levels. Data from three independent biological replicates were analyzed using the comparative method ΔΔCt and significance was assessed by two-tailed unpaired Student's t-tests. Primer sequences for qPCR are listed in Table S4.

### Overexpression of *siah-1* by CRISPR activation system

Overexpression of *siah-1* was achieved using a CRISPR activation system, employing dCas9::VP64 and promoter-specific sgRNAs as previously described (31). In brief, sgRNA target sequences specific for the promoter of *siah-1* were inserted into L4440_BioBrick-sgRNA (Addgene, plasmid #177783), designed as sgRNA *siah-1* A and sgRNA *siah-1* B. E.coli HT115 bacteria harboring vectors expressing sgRNA *siah-1* A, sgRNA *siah-1* B and the control vector L4440_BioBrick-SCR-sgRNA-B (Addgene, plasmid #177812) were firstly cultured on LB agar plates supplemented with 100 μg/ml ampicillin and 12.5 μg/ml tetracycline, and then single colonies inoculated into liquid LB medium containing 100 μg/ml ampicillin and grown overnight followed by centrifugation for 30 min at 3200*g* at 4 °C.The Bacterial pellets were resuspended and spotted on NGM plates containing 100 μg/ml ampicillin and 1 mM IPTG. Spotted bacteria were allowed to grow for 24 h before use. *C. elegans* strains carrying the *risIs33*(dCas9::VP64) transgene were cultured on the prepared plates for phenotype analysis after assessing the expression of *siah-1 via* qPCR or western blot. The specific sgRNA target sequences for *siah-1* promoter are provided in Table S4.

### Western blot analysis

For large-scale experiments, synchronized worms were collected in 1.5 ml sterile tubes and washed several times in M9 buffer before being frozen in liquid nitrogen. Worm lysates were prepared by sonication using the Bioruptor Plus Sonication System in a nondenaturing lysis buffer [50 mM Tris-HCl (pH 8.0), 150 mM NaCl, 1 mM EDTA, 1% Triton X-100, and 1 mM PMSF]. For small scale experiments, 50 to 300 worms were picked into 30∼50 μl lysis buffer and frozen in liquid nitrogen before grinding to obtain homogenized solutions. Prior to Western blotting, samples were mixed with 5 × SDS loading buffer, thoroughly vortexed, and denatured at 100 °C for 10 min. Proteins were then resolved by SDS-PAGE and analyzed by Western blot using specific antibodies.

### Antibodies

Polyclonal antibodies against DVE-1 were generated in the rat by immunization with a purified C-terminal 6xHis-tagged fragment of DVE-1 (amino acids 153–392) expressed in *E. coli* BL21(DE3). Antibodies against *C. elegans* SIAH-1 were obtained from rats immunized with GST-SIAH-1 (ΔE3) fusion protein. Sera were collected from rat blood by centrifugation at 15,000*g*, 4 °C, aliquoted and stored in 0.02% NaN_3_ at −80 °C before use. These two antibodies were tested and found effective, and they were used at 1:800 dilution for Western blot analysis. Commercial antibodies purchased and diluted for western blot analysis were as follows: mouse anti-GFP antibody (#66002-1-lg, Proteintech) 1:1500, mouse anti-Actin antibody (#A00702-100, GenScript) 1:3000, mouse anti-Aβ antibody (BioLegend, 6E10; Cat#803001), 1:2000, mouse anti-Myc-tag (#SC-40, SANTA CRUZ BIOTECHNOLOGY) 1:2000, mouse anti-Ubiquitin (#sc-8017, SANTA CRUZ BIOTECHNOLOGY) 1:1000, rat anti-HA (Roche, 11867423001) 1:2000, HRP-conjugated goat anti-mouse IgG (#115-035-174, Jackson ImmunoResearch), HRP-conjugated goat anti-rat IgG (#31470, Thermo scientific).

### Immunoprecipitation in *C. elegans*

Synchronized L1 stage worms were cultured on 90 mm NGM plates until L4 stage. These worms were then transferred to M9+OP50 medium with or without 10 μM MG132 for at least 4 h at 20 °C. These worms were then collected and washed several times with M9 buffer before being resuspended in lysis buffer [50 mM Tris-HCl (pH 7.6), 150 mM NaCl, 0.5 mM EDTA, 1% NP40, 0.5% Tween 20] containing protease inhibitor cocktail and frozen in liquid nitrogen. After thawing, worms were homogenized at 4 °C using a tissue grinder (Servicebio, KZ-III-F) and then centrifuged at 15,000*g* for 20 min at 4 °C to remove debris. The supernatant was collected and incubated with GFP-Trap Agarose (ChromoTek, gta) overnight at 4 °C, and the beads were centrifuged and washed three times with wash buffer [50 mM Tris-HCl (pH 8.0), 300 mM NaCl], with the last wash buffer discarded completely. The beads were subsequently resuspended in an equal volume of 2 × SDS loading buffer containing 5% 2-mercaptoethanol, then boiled at 100 °C for 10 min. Samples were then analyzed by Western blot.

### Mammalian cell culture and plasmid transfection

HEK293T cells were cultured in Dulbecco’s modified Eagle’s medium (DMEM, #SH30022.01, HyClone) supplemented with 10% fetal bovine serum (FBS, #SV30208.02, HyClone), 100 U/ml penicillin, and 100 μg/ml streptomycin at 37 °C under a 5% CO_2_ atmosphere. The authenticity of HEK293T cells was validated using STR profiling, and were free from *mycoplasma* contamination for all experiments. Plasmids were transfected into cultured cells facilitated with the PEIMAX reagent (#24765-1, Polysciences) according to the manufacturer’s instructions. The following plasmid amount were used for different culture vessels: 2 μg per well for the six-well plate, 5 μg per 60 mm dish, and 10 μg per 100 mm dish.

### GST-pull down

Recombinant proteins of GST-SIAH-1(ΔE3), GST-SIAH-1(1-191), and GST-SIAH-1(192-419) were expressed in *E. coli* BL21(DE3) bacterial cells and induced with 0.1 mM IPTG at 16 °C for 16 h. Proteins were purified using glutathione-Sepharose beads and eluted with elution buffer containing 25 mM Tris-HCl (pH 8.0), 150 mM NaCl, 3 mM DTT plus 15 mM reduced glutathione (GSH).

Full-length DVE-1, truncated DVE-1(1-178) and DVE-1(179-468) with N-terminal Myc tags were generated by cloning the corresponding coding sequences into the pCAG-Myc vector and expressed in HEK293T cells. Cells were transfected with 10 μg of plasmid DNA per 100 mm dish and harvested 48 h post-transfection.

For pull down assays, 5 μg of purified GST or GST-SIAH-1(ΔE3), GST-SIAH-1(1-191), and GST-SIAH-1(192-419) proteins immobilized on glutathione-Sepharose beads were incubated with cell lysates containing Myc-DVE-1, truncated Myc-DVE-1(1-178) or Myc-DVE-1(179-468). Beads were washed five times with washing buffer [50 mM Tris-HCL (pH 7.6), 250 mM NaCl, 0.5% NP-40, 1 mM EDTA]. Bound proteins were eluted, separated by SDS-PAGE, and visualized by autoradiography.

### Chemical treatments for worms

Synchronized worms expressing DVE-1::GFP were cultured on NGM plates seeded with *E. coli* OP50 from hatching till day 1 of adulthood. Worms were then transferred to M9 buffer containing OP50 bacteria and treated with DMSO (control), 10 μM MG132 (proteasomal inhibitor) (Sigma-Aldrich), 20 mM NH_4_Cl (lysosomal inhibitor) (Sigma-Aldrich), or bafilomycin A1 (2 μM or 4 μM) (lysosomal inhibitor) (Sigma-Aldrich) at 20 °C for 4 h. Following treatment, worms were washed and collected for sonication to generate homogenized solutions, Western Blot were then used to test the DVE-1-GFP level using GFP antibody.

### Ubiquitination of DVE-1 *in vivo*

To examine *in vivo* ubiquitination of DVE-1, synchronized young adult worms expressing either GFP alone or DVE-1::GFP were collected and washed in M9 buffer, then they were incubated with lysis buffer [50 mM Tris-HCl (pH 7.6), 150 mM NaCl, 0. 5 mM EDTA, 1% NP40, 0.5% Tween 20] supplemented with protease inhibitor cocktail, completely homogenized at 4 °C using a tissue grinder (Servicebio, KZ-III-F), and centrifuged at 15,000*g* for 20 min at 4 °C to remove debris. Samples were then incubated with GFP-Trap beads overnight at 4 °C, washed three times with washing buffer [50 mM Tris–HCl (pH 7.6), 300 mM NaCl], and subjected to Western blot to detect the ubiquitin levels using ubiquitin antibody.

In addition, for strains expressing HA-UBQ-2; DVE-1::GFP, HA::UBQ-2-K48R; DVE-1::GFP, or HA::UBQ-2-K63R; DVE-1::GFP, ubiquitination levels were determined by immunoprecipitation with an anti-GFP antibody followed by Western blotting with an anti-HA antibody.

### Paralysis assays

*C. elegans* strains used for paralysis analysis were maintained at 15 °C on NGM plates seeded with OP50 bacteria. Synchronized L4 stage worms were transferred to 25 °C for culture and analysis. Paralysis was assessed daily starting 24 h after temperature shift by gently touching the animal’s head with a platinum wire. Worms that were able to move the head but not the rest of the body were considered paralyzed.

### Lifespan assays

Lifespan experiments were performed at 20 °C. For one strain, 150 synchronized L4 stage worms of each strain (∼50 worms/plate) were prepared and scored as day 0, then they were scored and transferred to fresh plates every 2 days during the fertility period. After 1 week, worms were scored daily for viability and transferred to fresh plates and every 3 or 4 days. Worms were scored as dead if they did not respond to gentle touches with a platinum wire worm pick on the body. Worms that had internally hatched larvae or crawled off the agar surface were censored.

### MitoTracker staining

The MitoTracker Red CMXROS (Molecular Probes) was reconstituted in DMSO to prepare a 1 mM stock solution. This stock was then diluted to a final concentration of 1 μM in a suspension of OP50 bacteria before being spread onto NGM plates. Approximately 100 Eggs from each strain were placed on the on the MitoTracker-containing NGM plates and allowed to develop at 15 °C until they reached the L4 larval stage. Synchronized L4 worms from each strain were then transferred to 25 °C for further culture. Confocal images were captured after 24 h of growth.

### Statistical analysis

All experiments were performed with at least three biological replicates, yielding consistent or comparable results. Data were analyzed using Excel (Microsoft Office) or Graphpad Prism 9.0.0 software to generate curves or bar graphs. Error bars in each column graph represent the mean and standard deviation (SD), and descriptive statistics are detailed in the figure legends where applicable.

## Data availability

All data supporting the findings of this study are available within this paper and its supporting information.

## Supporting information

This article contains supporting information. Figures S1–S5, Tables S1–S4, Supplementary excel file 1 related to Figure 2, Supplementary excel file 2 related to metadata for all quantitative experiments.

## Conflict of interest

The authors declare that they have no conflicts of interest with the contents of this article.

## Figures and Tables

**Figure S5:**