# Supplementary material for: Inactivation of SIAH-1 E3 ligase attenuates Aβ toxicity by suppressing ubiquitin-dependent DVE-1 degradation in Caenorhabditis elegans models of Alzheimer’s disease
Source: J Biol Chem. 2025 May 9;301(6):110226. doi: 10.1016/j.jbc.2025.110226 (PMC12179603; doi:10.1016/j.jbc.2025.110226)
Supplement: Supplemental legends [file mmc1.docx]

**Supplementary figure legends:**

**Figure S1. Aβ induces UPR^mt^** **and impairs proteasome function (related to Figure 1)**. (A) Representative fluorescence images of *hsp-60*p::GFP in WT and *dvIs2* young adult worms. Scale bar: 100 μm. Quantification showed increased *hsp-60*p::GFP fluorescence in *dvIs2* worms compared to WT controls (n ≥ 15 per group; unpaired t-tests; ****P < 0.0001). (B) Transcript levels of *hsp-6* and *hsp-60* were examined by qPCR in day 1 adult wild type and *dvIs2* worms (three experiments; ***P < 0.001). (C) The mRNA levels of the UPR^mt^-related genes, including *clpp-1*, *flp-2*, *haf-1*, *atfs-1*, *dve-1*, *ubl-5*, *jmjd-1.2* and *jmjd-3.1,* were examined by qPCR in wild-type and *dvIs2* animals at day 1 stage (three experiments; ns, not significant, *P < 0.05, **P < 0.01, ***P < 0.001, ****P < 0.0001). (D) Representative fluorescence images of *mgIs72* and *dvIs2; mgIs72* worms at day 1 and day 4 stages. Scale bar: 100 μm. *rpt-3*p::GFP fluorescence was quantified and showed significant increases in Aβ-expressing worms (n ≥ 15 per group; **P < 0.01, ****P < 0.0001). (E) Representative fluorescence images of DVE-1::GFP in *zcIs39* and *dvIs2; zcIs39* worms at day 1 and day 4 stages. Scale bar: 100 μm. Quantification showed reduced DVE-1::GFP levels in Aβ-expressing worms (n ≥ 15 per group; *P < 0.05, **P < 0.01, ****P < 0.0001).

**Figure S2. Expression of *siah-1* or *dve-1* was accessed (related to Figure 2).** (A) *siah-1* transcript levels were analyzed by qPCR in wild-type and *dvIs2* animals at L4, day 1, and day 2 stages. Statistical significance was determined by unpaired Student’s t-tests (*P < 0.05, **P < 0.01). (B) Western blot analysis of SIAH-1 and β-actin (loading control) in *dvIs2; zcIs39* worms treated with EV and *siah-1* RNAi. SIAH-1 protein levels (normalized to β-actin) were quantified across developmental stages (L4, day 1, day 2). (C) *dve-1* mRNA levels were examined by qPCR in EV and *siah-1* RNAi animals at day 1 stage (three experiments; ns, not significant). (D) Western blot analysis of SIAH-1 and β-actin (loading control) in *zcIs39* worms treated with EV and *siah-1* RNAi. SIAH-1 protein levels were quantified as in (B). (E) Relative *siah-1* expression in *risIs33*(dCas9::VP64) worms fed with HT115 bacteria carrying sgRNA SCR, sgRNA *siah-1* A or sgRNA *siah-1* B, as determined by qPCR (three experiments; *P < 0.05). (F) Relative *dve-1* expression in *risIs33*(dCas9::VP64) worms fed with HT115 bacteria carrying sgRNA SCR, sgRNA *siah-1* A or sgRNA *siah-1* B, as analyzed by qPCR (three experiments; ns, not significant, *P < 0.05).

**Figure S3. Lysosome is not involved in DVE-1 degradation and UBC-25 contributes to the degradation of DVE-1 (related to Figure 3).** (A) Schematic representation of *siah-1* genomic DNA. Exons (solid bars) and introns (lines) are depicted. Deletion sites for the *tm1968* and *syb4782* alleles are indicated. Scale bar: 150bp. (B) PCR confirmation of the *tm1968* and *syb4782* deletion sites. (C) SIAH-1 was examined in N2 and *siah-1(tm1968)* mutant animals by Western blot, and *tm1968* is predicted to be a null allele. (D) Representative DIC and fluorescence micrographs of DVE-1::GFP in wild type and *siah-1(tm1968)* worms, Scale bar: 100 μm. DVE-1::GFP fluorescence was quantified and showed significant increases in *siah-1(tm1968)* worms (n ≥ 15 per group; ***P < 0.001). (E-F) Western blot analysis of DVE-1::GFP levels in day 1 adult *syb1984* worms treated with DMSO or 20mM NH_4_Cl (F) Western blot analysis of DVE-1::GFP after treatment with DMSO (control), 2 μM or 4 μM Bafilomycin A1 (BafA1). (G-H) Western blot analysis of DVE-1::GFP and β-actin in *zcIs39* (G) and *syb1984* (H) strains treated with EV or *rab-7* RNAi. (I) DVE-1::GFP and β-actin were examined by Western blot in *zcIs39* and *scav-3(ok1286); zcIs39* animals at day 1 stage. (J) Western blot analysis of DVE-1::GFP and β-actin in day 1 adult *syb1984* and *scav-3(ok1286); syb1984* animals. (K) *uba-1* RNAi efficiency was determined by qPCR. ****P < 0.0001 by t-test. (L) qPCR analysis of E2 enzyme-related gene expression. Unpaired t-tests, *P < 0.05, **P < 0.01, ***P < 0.001, ****P < 0.0001. (M) Co-IP analysis of the interaction between UBC-2 and SIAH-1. HEK293 cells were transfected with UBC-2-Myc and FLAG-SIAH-1 expression plasmids. Cell lysates were prepared 48 hours after transfection, IP with anti-FLAG or control IgG antibodies, and immunoblotted with anti-Myc or anti-FLAG antibodies. IgG-L: light chains of anti-FLAG or normal IgG.

**Figure S4. Truncated SIAH-1 interacts with DVE-1 (related to Figure 4).** (A) Purified GST or GST- SIAH-1 (ΔE3) was incubated with cell lysate expression of Myc-tagged DVE-1. Bound proteins were pulled down with glutathione-Sepharose beads and analyzed by Western blotting with anti-Myc antibodies. (B) Purified GST, GST- SIAH-1(1-191) or GST- SIAH-1(192-419) was incubated with cell lysate expression of Myc-tagged DVE-1 and then detected by Western blotting with anti-Myc antibodies after pull-down.

**Figure S5. Loss of SIAH-1 or overexpression of DVE-1 inhibits Aβ aggregation (related to Figures. 6-7).** (A) Schematic summary of DVE-1 degradation through the UPS pathway. E1: UBA-1, E2: UBC-25, E3: SIAH-1. (B) Assessment of paralysis in *dvIs2*, *dvIs2; zcIs39*, *dvIs2; siah-1(syb4782)*, and *dvIs2; siah-1(syb4782); zcIs39* worms after transfer to 25℃. Data represent the mean ± SD of three independent experiments (n=50 per experiment). Comparison at each time point was made using unpaired t-tests. *P < 0.05, **P < 0.01, ***P < 0.001, ****P < 0.0001. ns, not significant. (C) Analysis of paralysis in *dvIs2(unc-54p::*Aβ*)* and *dvIs2; siah-1(syb4782)* worms treated with EV or *ubc-25* RNAi after transfer to 25℃. Data represent the mean ± SD of three independent experiments (n= 50 per experiment). Statistical comparisons at each time point were performed using unpaired t-tests. *P < 0.05, **P < 0.01, ***P < 0.001, ****P < 0.0001, ns: not significant. (D) Analysis of paralysis in *dvIs2* and *dvIs2; zcIs39* worms treated with EV or *ubc-25* RNAi after transfer to 25℃. Data represent the mean ± SD of three independent experiments (n = 50 per experiment). Statistical comparisons at each time point were made using unpaired t-tests. *P < 0.05, **P < 0.01, ***P < 0.001, ****P < 0.0001, ns: not significant. (E) Survival analysis in *bkIs10*(aex-3p::h4R1NtauV337M), *bkIs10; syb4782* and *bkIs10; zcIs39* worms. See **Table S2** for survival statistics. **P < 0.01, ****P < 0.0001, log-rank test. (F) The Percentage of uncoordinated worms was significantly decreased in *bkIs10; siah-1(syb4782)* and *bkIs10; zcIs39* worms compared to *bkIs10* controls across different stages (n≥90 for each strain; unpaired t-tests; *P < 0.05, ***P < 0.001, ****P < 0.0001).

**Table S1: E2-related genes RNAi screen in *zcIs39***

**Table S2: Lifespan analysis**

**Table S3: List of *C. elegans* strains used in this study**

**Table S4: Primers used in this study**

**Supplementary excel file 1: mRNA-seq results, related to Figure. 2**

**Supplementary excel file 2: Metadata for quantitative experiments**
