# Supplementary material for: Inactivation of SIAH-1 E3 ligase attenuates Aβ toxicity by suppressing ubiquitin-dependent DVE-1 degradation in Caenorhabditis elegans models of Alzheimer’s disease
Source: J Biol Chem. 2025 May 9;301(6):110226. doi: 10.1016/j.jbc.2025.110226 (PMC12179603; doi:10.1016/j.jbc.2025.110226)
Supplement: Table S1 [file mmc4.docx]

**Table S1: E2-related gene RNAi screen in *zcIs39(dve-1::gfp)***

| Gene name | Sequence name | Source | RNAi in *zcIs39* (3 independent results) | | |
| --- | --- | --- | --- | --- | --- |
| EV (control) | L4440 | Yang lab | - | - | - |
| *ubc-1* | *C35B1.1* | RNAi library | - | - | - |
| *ubc-2/let-70* | *M7.1* | RNAi library | + | + | + |
| *ubc-3* | *Y71G12B.15* | This study | - | - | - |
| *ubc-6* | *D1022.1* | RNAi library | - | - | - |
| *ubc-7* | *F58A4.10* | RNAi library | - | - | - |
| *ubc-8* | *Y94H6A.6* | RNAi library | - | - | - |
| *ubc-9* | *F29B9.6* | RNAi library | + | - | - |
| *ubc-12* | *R09B3.4* | RNAi library | - | - | - |
| *ubc-13* | *Y54G2A.31* | This study | - | - | - |
| *ubc-14* | *Y87G2A.9* | This study | - | - | - |
| *ubc-15* | *Y110A2AR.2* | RNAi library | + | - | - |
| *ubc-16* | *Y54E5B.4* | RNAi library | - | - | - |
| *ubc-17* | *B0403.2* | RNAi library | - | - | - |
| *ubc-18* | *R01H2.6* | RNAi library | - | - | - |
| *ubc-19* | *Y69H2.6* | RNAi library | - | - | - |
| *ubc-20* | *F40G9.3* | RNAi library | - | - | - |
| *ubc-21* | *C06E2.3* | RNAi library | - | - | - |
| *ubc-22* | *C06E2.7* | RNAi library | - | - | - |
| *ubc-23* | *C28G1.1* | RNAi library | - | - | - |
| *ubc-24* | *F49E12.4* | RNAi library | - | - | - |
| *ubc-25* | *F25H2.8* | RNAi library | ++ | + | ++ |
| *ubc-26* | *Y110A2AM.3* | RNAi library | + | - | - |

Most of the RNAi clones were derived from the Ahringer Genomic RNAi library, referred to as RNAi library. Fluorescence of DVE-1::GFP in EV RNAi was denoted as”-” and “+ “means enhanced expression. “++” indicates a significant increase in fluorescence intensity.
