# Supplementary material for: Inactivation of SIAH-1 E3 ligase attenuates Aβ toxicity by suppressing ubiquitin-dependent DVE-1 degradation in Caenorhabditis elegans models of Alzheimer’s disease
Source: J Biol Chem. 2025 May 9;301(6):110226. doi: 10.1016/j.jbc.2025.110226 (PMC12179603; doi:10.1016/j.jbc.2025.110226)
Supplement: Table S2 [file mmc5.docx]

**Table S2: Lifespan analysis**

| Figures | Strains | Median  (days) | Mean±SD  (days) | N | P-value | Survival Increase (%) |
| --- | --- | --- | --- | --- | --- | --- |
| Figure 6C | N2 | 20 | 19.57±5.52 | 145 |  |  |
|  | *dvIs2* | 6 | 7.60±4.83 | 144 | ^****^P<0.0001 | -61.17% |
|  | *dvIs2; zcIs39* | 16 | 14.95±6.02 | 150 | ^****^P<0.0001 | 96.71% |
| Figure 6D | N2 | 18 | 17.87±5.64 | 145 |  |  |
|  | *dvIs2* | 6 | 7.35±4.85 | 149 | ^****^P<0.0001 | -58.87% |
|  | *dvIs2; syb4782* | 11 | 11.36±4.32 | 151 | ^****^P<0.0001 | 54.56% |
| Figure 6E | *gnaIs2* | 14.5 | 14.69±5.42 | 142 |  |  |
|  | *gnaIs2; syb4782* | 19 | 18.10±5.15 | 142 | ****P<0.0001 | 23.21% |
|  | *gnaIs2;zcIs39* | 18 | 17.48±6.26 | 146 | ****P<0.0001 | 18.99% |
| Figure S4E | *bkIs10* | 12 | 11.09±4.68 | 141 |  |  |
|  | *bkIs10; syb4782* | 13 | 13.12±3.12 | 139 | ^**^P=0.0033 | 18.30% |
|  | *bkIs10; zcIs39* | 14 | 13.14±4.39 | 136 | ^****^P<0.0001 | 18.49% |
