# Supplementary material for: Inactivation of SIAH-1 E3 ligase attenuates Aβ toxicity by suppressing ubiquitin-dependent DVE-1 degradation in Caenorhabditis elegans models of Alzheimer’s disease
Source: J Biol Chem. 2025 May 9;301(6):110226. doi: 10.1016/j.jbc.2025.110226 (PMC12179603; doi:10.1016/j.jbc.2025.110226)
Supplement: Table S3 [file mmc6.docx]

**Table S3: List of *C. elegans* strains used in this study**

| Strain name | Genotype | Source |
| --- | --- | --- |
| N2 | *C. elegans* wild type | CGC |
| CK10 | *bkIs10 (aex-3p::*h4R1NtauV337M *+ myo-2p::*GFP*).* | CGC |
| CL2006 | *dvIs2[unc-54p::*Aβ_1-42_*, rol-6(su1006)]* | CGC |
| GR2183 | *mgIs72 [rpt-3p::*GFP *+ dpy-5(+)]* II | CGC |
| GRU102 | *gnaIs2 [myo-2p::*YFP *+ unc-119p::Abeta1-42]* | CGC |
| LSD2104 | *xchIs15[hsp16.2p*::ssSel1::FLAG::sfGFP::spacer::hAβ_1-42_::*let-858 3’UTR + rol-6(su1006)].* | CGC |
| MIR249 | *risIs33[K03A1.5p::*3xFLAG::SV40-NLS::dCas9::SV40-NLS::VP64::HA *+ unc-119(+)].* | CGC |
| RB1481 | *ubc-25(ok1732)* I. | CGC |
| SJ4058 | *zcIs9 [hsp-60::*GFP *+ lin-15(+)]* V | CGC |
| SJ4100 | *zcIs13 [hsp-6p::*GFP *+ lin-15(+)].* | CGC |
| SJ4197 | *zcIs39(dve-1p::dve-1::gfp)* II | CGC |
| PHX1984 | *dve-1(syb1984[dve-1::gfp])* X | Tian lab |
| SNU22 | *ced-1::flag(xwh17); ha::ubq-2(xwh20)* | Xiao Lab |
| SNU23 | *ced-1::flag(xwh17); ha::ubq-2-K48R(xwh23)* | Xiao Lab |
| SNU24 | *ced-1::flag(xwh17); ha::ubq-2-K63R(xwh24)* | Xiao Lab |
|  | *yqIs179 (P_Y37A1B.5_ tomm-20::mCherry)* 4x | Yang Lab |
| PHX4782 | *siah-1(syb4782)* IV 6x | SunyBiotech |
| WSX54 | *dvIs2[unc-54p::*Aβ_1-42_*, rol-6(su1006)]; zcIs39(dve-1::gfp)* | This study |
| WSX84 | *bkIs10(aex-3p::h4R1NtauV337M + myo-2p::GFP); zcIs39(dve-1::gfp)* | This study |
| WSX117 | *dvIs2[unc-54p::*Aβ_1-42_*, rol-6(su1006)]; siah-1(syb4782)* | This study |
| WSX120 | *siah-1(syb4782); zcIs39(dve-1::gfp)* | This study |
| WSX163 | *dvIs2; zcIs39(dve-1::gfp); siah-1(syb4782)* | This study |
| WSX195 | *dvIs2[unc-54p::*Aβ_1-42_*, rol-6(su1006)];*  *zcIs9(hsp-60p::gfp)* | This study |
| WSX196 | *siah-1(syb4782); dve-1(syb1984[dve-1::gfp])* X | This study |
| WSX197 | *dvIs2[unc-54p::Aβ_1-42_, rol-6(su1006)]; dve-1(syb1984)* | This study |
| WSX212 | *xwh20; dve-1(syb1984[dve-1::gfp])* X | This study |
| WSX213 | *xwh23; dve-1(syb1984[dve-1::gfp])* X | This study |
| WSX214 | *xwh24; dve-1(syb1984[dve-1::gfp])* X | This study |
| WSX250 | *siah-1(tm1968); zcIs39(dve-1p::dve-1::gfp)* II | This study |
| WSX252 | *scav-3(ok1286); zcIs39(dve-1p::dve-1::gfp)* II | This study |
| WSX253 | *scav-3(ok1286); dve-1(syb1984[dve-1::gfp])* X | This study |
| WSX254 | *siah-1(tm1968)* IV 4x | This study |
| WSX259 | *fzo-1(tm1133) II; zcIs13(hsp-6p::gfp); risIs33*(dCas9::VP64) | This study |
| WSX267 | *ubc-25(ok1732)* I*; zcIs39(dve-1p::dve-1::gfp)* II | This study |
| WSX268 | *ubc-25(ok1732)* I*; dve-1(syb1984[dve-1::gfp])* X | This study |
| WSX286 | *siah-1(syb4782); xchIs15* | This study |
| WSX295 | *siah-1(syb4782); mgIs72(rpt-3p::gfp)* | This study |
| WSX296 | *zcIs39(dve-1p::dve-1::gfp)* II*; xchIs15* | This study |
| WSX349 | *dvIs2; yqIs179(tomm20::mcherry)* | This study |
| WSX364 | *bkIs10(aex-3p::hTauV337M+myo-2p::gfp); syb4782* | This study |
| WSX396 | *dvIs2; siah-1(syb4782); yqIs179(tomm-20::mCherry)* | This study |
| WSX397 | *dvIs2; zcIs39(dve-1::gfp); yqIs179(tomm-20::mCherry)* | This study |
| WSX401 | *gnaIs2[myo-2p::*YFP *+ unc-119p::Abeta1-42]*; *zcIs39* | This study |
| WSX402 | *gnaIs2; siah-1(syb4782)* | This study |
