# Supplementary material for: Inactivation of SIAH-1 E3 ligase attenuates Aβ toxicity by suppressing ubiquitin-dependent DVE-1 degradation in Caenorhabditis elegans models of Alzheimer’s disease
Source: J Biol Chem. 2025 May 9;301(6):110226. doi: 10.1016/j.jbc.2025.110226 (PMC12179603; doi:10.1016/j.jbc.2025.110226)
Supplement: Table S4 [file mmc7.docx]

**Table S4: Primers used in this study**

| qPCR primers | |
| --- | --- |
| Primer name | **Sequences (5’-3’)** |
| *hsp-6* Fw | GAAGATACGAAGACCCAGAGGTTC |
| *hsp-6* Rv | CAACCTGAGATGGGGAATACACT |
| *hsp-60* Fw | AGGGATTCGAGAGCATTCGTCAAG |
| *hsp-60* Rv | TGTGGCGACTTGAGCGATCTCTTC |
| *clpp-1* Fw | AACAGTCCAGGCGGCAGTGT |
| *clpp-1* Rv | ACCACCACTCGGCTGGTGAA |
| *flp-2* Fw | AGCTTCAGCAATGGCAAAAC |
| *flp-2* Rv | CAGCGGATTGAATCGTTTTC |
| *haf-1* Fw | GTTAATCAGACTGCGATCGAGCG |
| *haf-1* Rv | GCTCTCCATATGTGATATGTCCCG |
| *atfs-1* Fw | GAGCTGGAAGCATGGCG |
| *atfs-1* Rv | GGTGGCATTCCGTACTCATC |
| *dve-1* Fw | TAGCAGCTCTTGGACATTCGAGCA |
| *dve-1* Rv | CGAATGGCAAGGGTTTCCAGTTGT |
| *ubl-5* Fw | CGAATCAAGTGCAATCCATC |
| *ubl-5* Rv | TCATTGGTAGTAGAGCTCG |
| *lonp-1* Fw | ACAACACCACCATCAGCAAC |
| *lonp-1* Rv | GCACAATGGCCATCATCGTA |
| *pdr-1* Fw | CACTGACACCTGCAACACAA |
| *pdr-1* Rv | AGAACAGAGGTTGACGAGCA |
| *pink-1* Fw | TGGCTCCATATCCGAATGCT |
| *pink-1* Rv | CATACCAACGTGCAGTGTGA |
| *cco-1* Fw | GCTCGTCTTGCTGGAGATGATCGTT |
| *cco-1* Rv | GGTCGGCGTCGACTCCCTTG |
| *cyc-1* Fw | GTGCCGTGGTTCAAGGAT |
| *cyc-1* Rv | TCCACGTCGTACAGAAGC |
| *nduo-1* Fw | AGCGTCATTTATTGGGAAGAAGAC |
| *nduo-1* Rv | AAGCTTGTGCTAATCCCATAAATGT |
| *jmjd-1.2* Fw | ATCCGAGGAGAAAGCAGCAC |
| *jmjd-1.2* Rv | GGAATGGAGGCTCAGATCCG |
| *jmjd-3.1* Fw | GTGACAATGAAACGACGTTG |
| *jmjd-3.1* Rv | TTCGCTTGAAGTAGAAGCAG |
| *siah-1* Fw | ATCGGAACGGTGGTGGAGGA |
| *siah-1* Rv | CTTGCGGAGCCGAATCGCTA |
| *uba-1* Fw | TTCTTGGAGGAGTTCGGCAT |
| *uba-1* Rv | CGCTCGTAGCAAGAAGTAGC |
| *ubc-1* Fw | CAGTCCTGGCTCAATTTCGG |
| *ubc-1* Rv | TACGGTCATCGTCTGCATCA |
| *ubc-2* FW | TCTGCCTTGACATTCTCCGT |
| *ubc-2* RV | CGTCTTGTAGATGCGTGCAA |
| *ubc-3* Fw | AAGCAGGTGGAAGAGTCGAA |
| *ubc-3* Rv | CCTCCTCTTCATCGTCGTCA |
| *ubc-6* Fw | TGCTGCCCAACTCAAGTTTC |
| *ubc-6* Rv | GGGACCGAGCTTGTTTCTTC |
| *ubc-7* Fw | GTTACGAACGTCCGGAAGAG |
| *ubc-7* Rv | GCATCAACGTTTGCAGGAGA |
| *ubc-8* Fw | TGCCTACGAGAATGGTGTGT |
| *ubc-8* Rv | GAGATCGTAGAGGGCTGTCC |
| *ubc-9* Fw | CCGACGGAACATTGAACCTC |
| *ubc-9* Rv | TGACGGGAAATCGTCCTTGA |
| *ubc-12* Fw | CCCGTTGTTAAGTGCCTGAC |
| *ubc-12* Rv | CGCGTCGTTGAAGTCCATAA |
| *ubc-13* Fw | CTCCATTTGCTGGTGGTGTC |
| *ubc-13* Rv | CCGGCGACCATTTATCCTTG |
| *ubc-14* Fw | TGTCAGCATGTTGGCTGAAC |
| *ubc-14* Rv | TTAGACTTCCGAAGCGGGAA |
| *ubc-15* Fw | CGGAACTCCACAGGATCAGA |
| *ubc-15* Rv | ATGGCCGATGAAACTTGTCG |
| *ubc-16* Fw | ACACCTGCTCTCAGTGTTCA |
| *ubc-16* Rv | CACTGTCGTCATGGAACCAC |
| *ubc-17* Fw | GGAATTCGTCGGCATCTCTG |
| *ubc-17* Rv | ACTCAGCAGCCAAGTAAGGT |
| *ubc-18* Fw | AACTGTGGAGTGAAGGCGTA |
| *ubc-18* Rv | TGGTGGCTTGAATGGGTAGT |
| *ubc-19* Fw | GAGCCGCTCGATGGTTTATG |
| *ubc-19* Rv | GACACTTGCAAGCCCATCTT |
| *ubc-21* Fw | ACGGGTCACACGGAAGTGTA |
| *ubc-21* Rv | GCCCTCTGGACCCTTGATGA |
| *ubc-20* Fw | CCATTCTCGCCACCAAATGT |
| *ubc-20* Rv | TGAATCGACAGGAGAACCGT |
| *ubc-22* Fw | ATCGAGGTAGACGAGGAGGT |
| *ubc-22* Rv | TCACTACCACCCAGAGCAAG |
| *ubc-23* Fw | TTTATGCAGGAGGCTCACCA |
| *ubc-23* Rv | GAGCGTACTCGACTCTCCAA |
| *ubc-24* FW | ACTTTGCTCGCCCATGCTAC |
| *ubc-24* RV | CACAGGACGTGAGAGATCCG |
| *ubc-25* Fw | ATGACGTTCCAGTCATCGGA |
| *ubc-25* Rv | CACTTCCACGACCTTCATCG |
| *ubc-26* Fw | CGCCCGCAATTCTTATGCTA |
| *ubc-26* Rv | AGCCCAGTGTCGGTTGATTA |
| *siah-1* Promoter-Specific sgRNA target sequences | |
| sgRNA name | **Target Sequences** |
| sgRNA *siah-1* A Fw1 | AGGGTATCCCGGTCAGTCATTCGT |
| sgRNA *siah-1* A Rv1 | AAACACGAATGACTGACCGGGATA |
| sgRNA *siah-1* A Fw2 | AGGGAGACCGACGACGAGATGTGT |
| sgRNA *siah-1* A Rv2 | AAACACACATCTCGTCGTCGGTCT |
| sgRNA *siah-1* B Fw1 | AGGGGAGAGAGAGAGAGAGACGGT |
| sgRNA *siah-1* B Rv1 | AAACACCGTCTCTCTCTCTCTCTC |
| sgRNA *siah-1* B Fw2 | AGGGGAGACCGACGACGAGATGTG |
| sgRNA *siah-1* B Rv2 | AAACCACATCTCGTCGTCGGTCTC |
| primers for mutant confirmation | |
| *syb4782* NS | GGCATCACACAACAGCAATG |
| *syb4782* CAS | GCAGCTCTGCATCATAACCC |
| *tm1968* NS | CGCAAGGAATCCAAAGCGTC |
| *tm1968* CAS | GCTGTGCAGCATTTGAATCG |
| *ok1286* NS1 | GGCGGGAGTTCTCTTGTTAATC |
| *ok1286* NS2 | CATTGTGGTCCCATAGGCG |
| *ok1286* CAS | CGTCGAATAGAGCATCGCTTAC |
| *ok1732* NS | CAGATACTGTCTGCCTCGGTTG |
| *ok1732* CAS1 | CGAACGGTGGATCACAAGGG |
| *ok1732* CAS2 | GTGAACACTCCATCGCGTG |
| HA-*ubq-2* NS | GTACCCATATGATGTCCCGG |
| HA-*ubq-2* CAS | CTTCTTTCTGCAGTTGCTGG |
| *tm1133* NS | GTCGGCATTCCCCTGATTCC |
| *tm1133* CAS | CATCGTGGACTTCACAGCTG |
| *risIs33* NS | GAGGACAACGAGCAAAAGCA |
| *risIs33* CAS | GAGGTTGGTGAGGGTGAAGA |
| primers for cloning | |
| FLAG-*siah-1* Fw | GATGACGATAAAGGATCCATGAGTAATCGGAACGGTG |
| FLAG-*siah-1* Rv | ctctagatgcatgCTCGAGCTAGTCGTACTCGAGATC |
| Myc-*ubc-2* Fw | gccatggaggccgaattcATGGCTCTCAAAAGAATCCAG |
| Myc-*ubc-2* Rv | gagtgcggccgcctcgagTCACATAGCGTACTTTTGCG |
| Myc-*ubc-25* Fw | ggccatggaggccgaattcATGGCGTGTCTTCGAAAAC |
| Myc-*ubc-25* Rv | gagtgcggccgcctcgagTTATCCTTCTGTTTTTGGAGG |
| GST-*siah-1* Fw | CTGGTTCCGCGTGGATCCATGAGTAATCGGAACGGTG |
| GST-*siah-1* Rv | CACGATGCGGCCGCTCGAGCTAGTCGTACTCGAGATC |
| GST-*siah-1*(1-191) Rv | CGATGCGGCCGCTCGAGCTACCGACAAGTCGGGC |
| GST-*siah-1*(192-419) Fw | CTGGTTCCGCGTGGATCCGGTCCAACTCCTTCTGTC |
| pCAG-Myc-*dve-1* Fw | gccatggaggccgaattcATGTTCCCAATGAGGGTAATC |
| pCAG-Myc-*dve-1*Rv | gagtgcggccgcctcgagTTATACGAAAACCTTCTGCTC |
| Myc-*dve-1*(1-178) Rv | gtgcggccgcctcgagTTAGCTTCTACGATCCTCGTTC |
| Myc-*dve-1*(179-468) Fw | gccatggaggccgaattcCCAACACAAGTACAAAG |
| *siah-1* RNAi Fw | gacggtatcgataagcttATGAGTAATCGGAACGGTGG |
| *siah-1* RNAi Rv | ggtggcggccgctctagaCTAGTCGTACTCGAGATCAG |
| *ubc-3* RNAi Fw | cgacggtatcgataagcttATGGACTCAAAAGCGTCAAC |
| *ubc-3* RNAi Rv | ggtggcggccgctctagaCTAATTTTCTCCTTGTCCCG |
| *ubc-13* RNAi Fw | gacggtatcgataagcttATGGCCGGGCAACTTCCGCG |
| *ubc-13* RNAi Rv | ggtggcggccgctctagaTCAGGCTTGAGCATAGTTC |
| *ubc-14* RNAi Fw | gacggtatcgataagcttATGGCTGGTTACGCTTTG |
| *ubc-14* RNAi Rv | gtggcggccgctctagaTTAGACTTCCGAAGCGGGAAG |
